# Decoding RNA Structural Ensembles: Energy Landscape Exploration of the TAR Stemloop

**DOI:** 10.1021/acs.jctc.5c01677

**Published:** 2026-01-05

**Authors:** Konstantin Röder

**Affiliations:** Randall Centre for Cell and Molecular Biophysics, 4616 King’s College London, London WC2R 2LS, United Kingdom

## Abstract

RNAs are key to understanding cellular mechanisms and a prime target for novel therapeutic interventions. However, RNA structural ensembles are notoriously difficult to study due to structural polymorphism and their highly dynamic nature, i.e., the fact that RNAs can adopt multiple structures and the transitions between them are fast. As a result, computational and experimental methods to study the RNA structure are limited in their usefulness, and often, a combination of multiple methods is required. Furthermore, RNA force fields are not yet developed to the same standard as those for proteins. Here, we demonstrate that energy landscape explorations via discrete path sampling enable a full mapping of structural ensembles and can capture mutational changes well. In this contribution, we show that the ensembles derived for the TAR stemloop and a derived stemloop (ES2) are sufficient to reproduce experimental observations without the need to introduce experimental data into the modeling beyond the force field parameters. Our modeling reveals significant complexity in both the structural ensemble and the activation pathway. Furthermore, we identify a transient binding pocket that emerges on the activation pathway.

## Graphical abstract

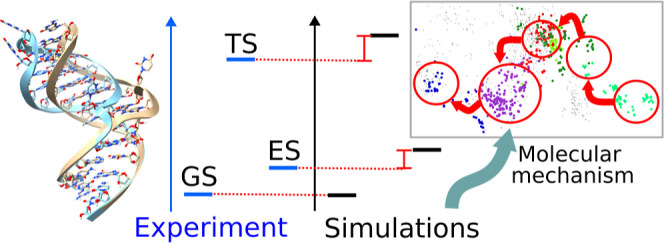

## 1 Introduction

Regulatory RNAs have emerged as a key class of biomolecules that control cellular function and determine,^1^ at least in parts, cellular activity.^2^ This role can only be adopted due to the existence of well-defined, stable 3D structures that are adopted by these RNAs. Analogous to proteins, the structure of RNAs directly impacts their function. However, RNAs do not exhibit a native structure as observed in globular proteins, but instead multiple competing states exist.^3^ This feature arises from the many noncanonical base pairing options that exist in RNAs, allowing for energetically similar but structurally different nucleotide interactions. As a result, RNA structure is best discussed as polymorphic, and therefore, a full structural description requires a consideration of the structural ensembles as opposed to a single native structure. This description must necessarily contain the following three elements:

1. The alternative structures.
2. The relative stability of distinct configurations (thermodynamics).
3. The transitions between structures (kinetics).

Perhaps not surprisingly, obtaining these details for any regulatory RNA is a significant challenge. For example, NMR spectroscopy is not yet capable of resolving the complex dynamics and interactions of RNAs, although significant progress is made in this field.^4,5^ Computational methods can fill this gap and aid in our understanding of regulatory RNAs. For example, SAXS experiments can be combined with computational approaches to gain valuable insights into RNA structures and dynamics.^6,7^

However, several issues need to be addressed in this context. First, RNA force fields are not yet of the same quality as those for proteins.^8–11^ This current deficiency is partly a consequence of the lack of experimental data. The consequences, especially for intermediate and metastable states, can be significant, leading to poor representation of noncanonical interactions and the stabilization of alternative structures.^10^ A second problem is that alternative structures require more sampling (i.e., more states need to be explored computationally), which may restrict the utility of common methods, such as molecular dynamics (MD) simulations. It is not straightforward to disentangle which of these effects is the more impactful. It has been suggested that reweighting computational ensembles with experimental constraints can significantly improve the overall quality of the structural ensemble.^12,13^ One of the most successful methods to describe RNA structural ensembles is based on this idea. FARFAR^14,15^ uses the known fragments of the RNA structure to assemble alternative RNA structures for a given sequence. A subsequent filtering of this library using NMR residual dipolar coupling (FARFAR-NMR)^16^ provides RNA structural ensembles, where the computed observables match experimental findings well. Nonetheless, there are a number of features that limit the current applicability of this approach. First, the number of known fragments is limited. Second, long-range interactions may not be represented well in fragment-based assemblies. Finally, experimental data for refinement are also limited in temporal resolution.

It is therefore worthwhile to consider whether computational work alone might be predictive using physics-based simulation techniques (such as MD) rather than data-driven methods. The key question in this context is returning to the balance between force field accuracy and sampling convergence. Given the current force fields and their inherent shortcomings, is it possible to sample RNA structural ensembles to obtain a good description of RNAs? In a first instance, a good description would be to match experimental findings, while a more ambitious aim would be predictive ensembles that enable the targeted design of experiments.

An overview of the field in 2018 ^17^ noted that one method, namely, the computational exploration of energy landscapes^18,19^ via discrete path sampling (DPS),^20,21^ was able to match experimental observations, though it noted that “there is currently limited experience with [its] application to nucleic acids.” Since then, further work with explicit energy landscape explorations for nucleic acids has been presented, all showing good agreement with experimental observables, for example, for the structural dynamics of the HP1 hairpin in 7SK RNA^22^ and the structural changes upon methylation of the ORF50 RNA from Kaposi’s sarcoma-associated Herpes virus.^23^

The method is explicitly designed to overcome the sampling problem by focusing on the geometric definitions of the transition states and local minima. This approach yields a faithful representation of the energy landscape and therefore the structural ensemble, while the computational cost is independent of barrier heights (as opposed to an exponential dependence for MD simulations).^24^ The use of implicit solvation introduces a limitation, which also impacts the representation of ions in the simulations. It is well established that explicit solvation models are more appropriate for the MD simulations of RNA,^17^ especially to reproduce local dynamics faithfully. However, we recently showed that the energy landscape framework can faithfully reproduce metastable/excited states in an implicit solvent.^10^ The improved sampling of higher energy configurations within the framework as compared to, for example, MD simulations, especially for dynamic molecules such as RNA, seems to be the key to reproduce global dynamics. This observation implies that local dynamics may not be accurately captured. This shortcoming can be overcome by combining the computational energy landscape framework with other computational techniques.^25^ This trade-off between good sampling and a less accurate local description is observed in this contribution, as well.

Despite previous work highlighting the utility of the approach, it has not been validated against systems with a large number of reference data. In particular, the method has not been benchmarked against the known dynamics in functional RNAs. Work so far focused mainly on solving problems that other methods could not satisfactorily answer. Here, a comprehensive analysis of the energy landscape for the HIV transactivation response element (TAR) is reported. Much data is available, both from computational and experimental approaches for this stemloop. TAR also exhibits complex dynamics, with at least one excited, biologically active state characterized. TAR is an RNA stemloop that contains two helical sections joined by a bulge. Both the bulge and the apical loop are highly dynamic,^26^ with distinct time scales for the two transitions highlighted by NMR spectroscopy.^27^ Changes in the RNA sequence impact this dynamic behavior and impact the protein interactions of TAR, which in turn are key for efficient transcription elongation of HIV.^28^ The apical loop dynamics yields an excited state (ES1) at around 13% occupancy and a lifetime of around 45 μs.^27^ A second excited structure (often referred to as ES2) relating to the bulge dynamics is much rarer but longer lived, with initial estimates of the occupation probability of 0.4% and 2 ms lifetime from NMR experiments.^29^

Subsequently, a hierarchical energy landscape was proposed,^30^ with a dynamic kinking motion of the RNA around the bulge at a time scale of ns to ms in the ground state and the transitions between the ground state (GS) and excited states (ES1 and ES2) at μs to ms. More recently, further work has confirmed these time scales using CEST NMR.^31^ Figure 1 illustrates the difference between the GS and ES2 states. While at least two excited states have been described, they are qualitatively different in their accessibility, as described above. The more populated excited state, which is based on loop dynamics, can be captured using classical MD simulation, though FARFAR-NMR provides better representations of the local dynamics around the ground state.^16^ The much bigger challenge is to accurately capture the bulge dynamics and the transition from the ground state to the ES2 state. Most of our analysis will focus on this transition as it meets the characteristics of a rare event.

**Figure 1:**
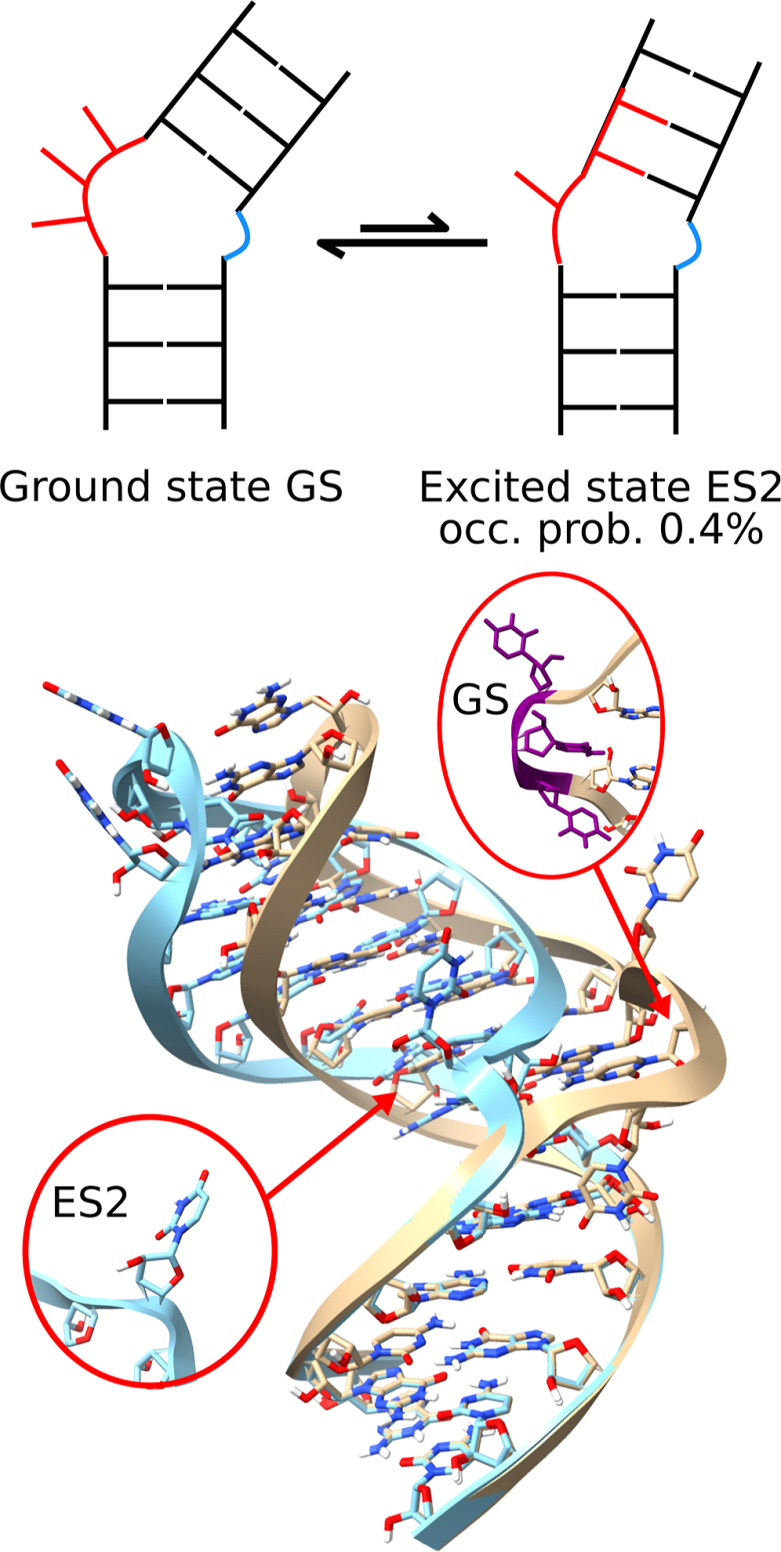
Schematic overview of the difference between GS and ES2 structures. The main change is the realignment of base pairing in the upper stem, leading to alterations in the bulge. This leads to a change in the orientation of the upper helical section, which stacks coaxially in ES2 (light blue), while the GS stacks do not follow this pattern (beige). For both structures, the bulge residues are shown. For GS, these are highlighted in purple.

The following criteria can be considered to be required for our exploration:

- Identify the excited states and correctly characterize their stability.
- Characterize the time scales of the transitions from GS to ES2 and vice versa correctly.
- Match known ensemble properties for the structure and derived observables.
- Yield detailed additional insight into the TAR structural ensembles.

Here, we demonstrate that the computational energy landscape framework can meet these criteria. For the first time, we show that the framework can reproduce experientially observed free energy barriers well for RNA. Furthermore, the complex transition pathways between ground and excited states can be captured in detail, including a detailed characterization of intermediates. In addition, we highlight how the structural ensembles captured by FARFAR-NMR provide a good representation of the ground state, but this purely computational approach is able to capture much more detail of the excited state sub-ensemble.

## 2 Results and Discussion

### 2.1 The Energy Landscape for TAR

The potential energy landscape for the TAR stemloop is shown in Figure 2, alongside key representative structures. The energy landscape is presented as a disconnectivity graph,^32,33^ where the *y*-axis is the energy and the *x*-axis is an arbitrary ordering, chosen to avoid any overlaps between the branches of the tree. Each branch is a local minimum, and branches merge when there is a path between them with transition states lower than the energy of the point where they merge. Broadly, the energy landscape exhibits two main funnels, each of which is further divided into subfunnels. The lowest potential energy structure is the ground-state (GS) structure. The funnels are well separated using the nucleobase distance for G10 and C21. This base pair is formed only in the excited state (ES2) and is indicated by red parts on the energy landscape. In line with previous work, the GS structure shows a closed loop, while the change in base pairing for the ES compacts the helix and opens up the loop.

**Figure 2:**
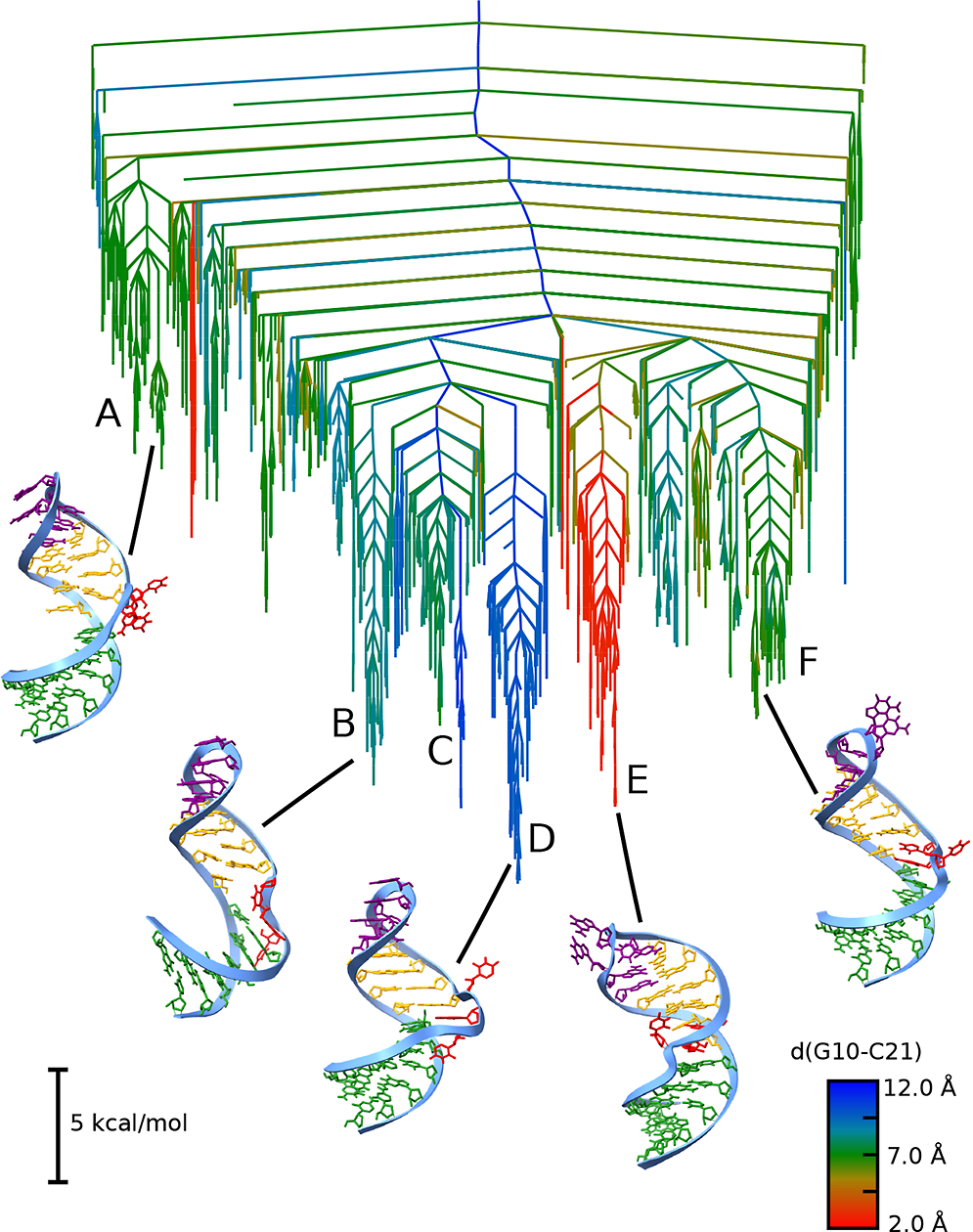
Potential energy disconnectivity graph for the TAR stemloop colored by the distance between G10 and C21. Red indicates close proximity (around 3 Å), indicating base pairing, while larger distance are shown in blue. The order parameter gives an idea of the distinct base pairing across the landscape. Example structures are shown, with the bottom six base pairs in the stem shown in green, the three bulge nucleotides U7, C8, and U9 in red, the next four stem base pairs in yellow, and the loop nucleotides in purple. The variation in base pairing and kinking between the top and bottom stems around the bulge is clearly visible. Six distinct funnels are indicated by the letters A to F, with D and E corresponding to the funnels containing the ground state and excited state ES2, respectively.

Alongside these structures, a range of different intermediates is found, with great variations in the base pairing around the bulge and significant changes in the kinking between the two helices on either side of the stem. These results already indicate a significant structural variety, which will be explored more in the next section.

A key question is how persistent the funnels indicated in the potential energy landscape are. To answer this question, we can consider the free energy landscape, as shown in Figure 3. The free energies are computed using harmonic superposition.^34^ The funnels observed in the potential energy landscape are persistent in the free energy landscape. A change observed is that there are some structures in funnel C that are similar or lower in free energy than those of the ground-state structures in funnel D. The difference between the structures lies in changes in base pairing in the bottom of the upper stem next to the bulge. This is likely a force field inaccuracy, with a G–U wobble base pair being formed over a Watson–Crick base pair. The time scale of the transition between these structures is much faster than toward the ES2 state. The formation of the G–U wobble is indeed observed on the actual pathway and discussed in more detail later on (see §2.3).

**Figure 3:**
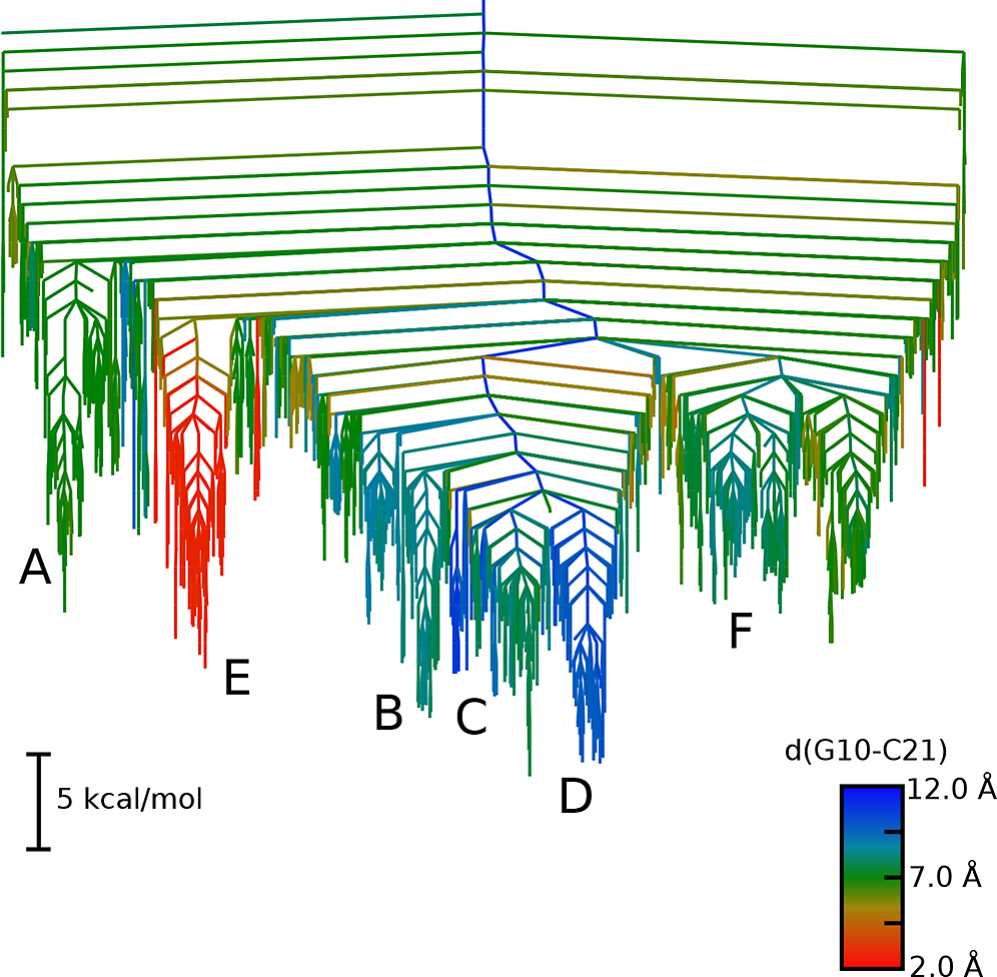
Free energy landscape for the TAR stemloop at 37 °C, using the same order parameter as Figure 2. The same funnels are labeled as in the potential energy landscape, showing that the selected funnels are important for both free and potential energy landscapes.

### 2.2 Structural Diversity of TAR

To characterize the structural ensemble and the diversity of features observed in the sub-ensembles, the minima are categorized by the funnels to which they belong on the energy landscape, as indicated in Figure 2. This assigns around 50% of minima to the six categories, with D containing the canonical ground state and E the excited state ES2. The minima not assigned are generally higher energy structures in intermediate regions between funnels.

A second categorization was attempted based on the dot-bracket representations of the secondary structure of the minima. A key issue identified with this structural approach was the presence of base pair wobbling, which meant that the secondary structure assignments used for dot-bracket representations were ambiguous. In many cases, the dot-bracket representations would not distinguish between a wobbling canonical base pair and the absence of a canonical base pair. For more fine grained approaches of structure classification, the classifications produced either too many categories or ambiguities in assignments, such as the XXX classification for nucleotide interactions in baRNAba.^35^ For this reason, such a structure-based classification was not pursued further.

The selection of sub-ensembles allows us to characterize important features of the structural ensemble of TAR. Figure 4 shows the representative base pairing for the ground and ES2 states. The data aligns with previous characterizations of both states, highlighting the shift in base pairs and the resulting change in bulge size from three nucleotides in the ground states to only one in the excited state. The lower stem is identical in both structures. The upper helical stem is shorter in the ground state but is formed of four canonical base pairs. The top base pair is noncanonical and less stable, explaining the known loop dynamics (GS to ES1). An alternative canonical base pair can be formed instead of the C–A base pair, which shortens the loop. This change is what has been described as the excited state (ES1) in the loop.^29^ On the potential energy landscape, the noncanonical base pair is lower in energy, but in the free energy, the canonical base pair is lowered. This transition is observed within funnels, indicating that the underlying energy function does not distinguish clearly between the canonical and noncanonical base pairs, likely due to the weaker stacking interactions if the canonical base pair is formed.

**Figure 4:**
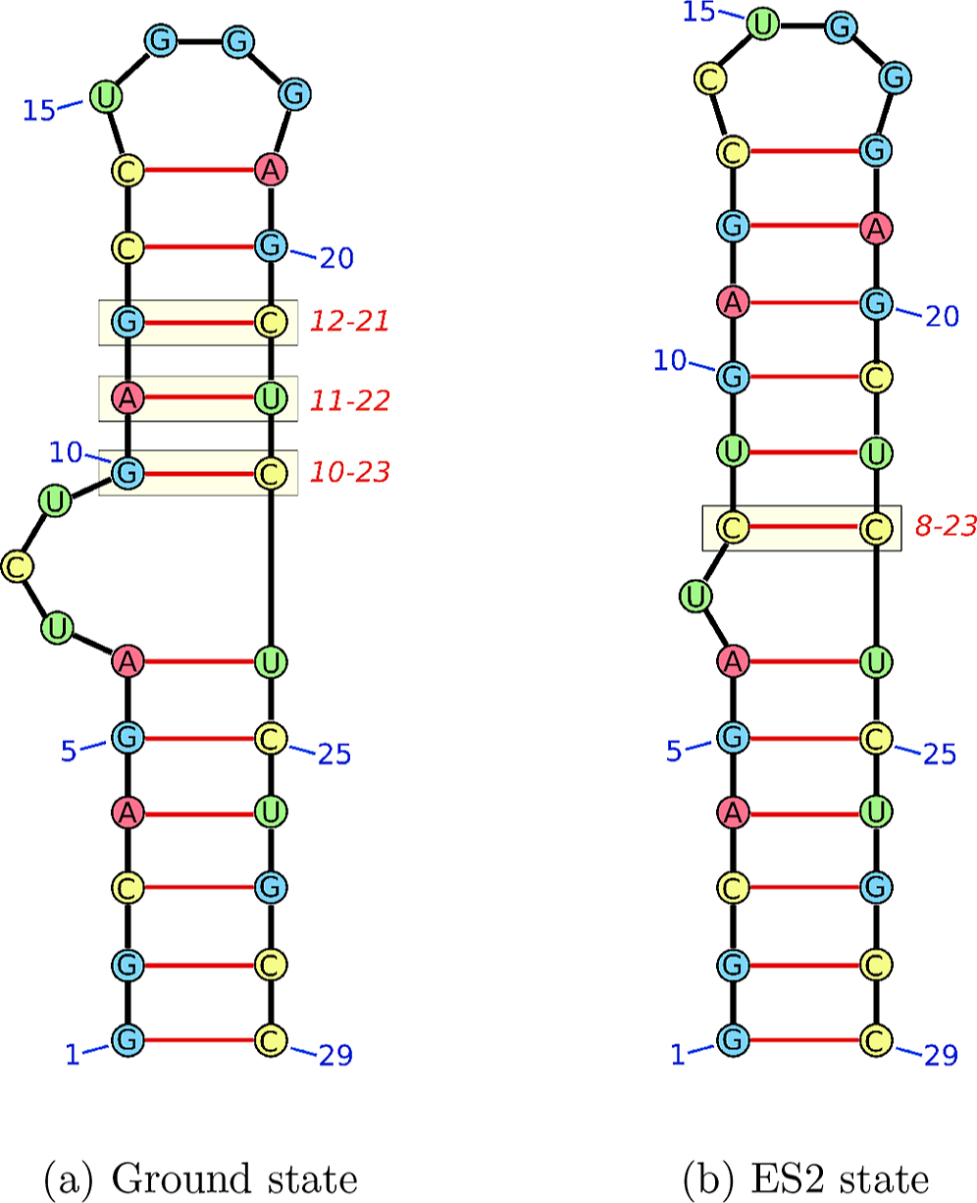
2D representations of RNA structure showing the base pairing for the structures funnels associated with the (a) ground state (D) and (b) ES2 state funnels (E). Base pairs were identified for all structures in each funnel using Barnaba.^35^ Key differences in base pairing are highlighted in boxes and labeled. The top of the loop for the ground state is shown in the stacked conformation (i.e., the noncanonical C–A base pair is formed).

The ES2 state in contrast relies on more noncanonical base pairs overall, with only two canonical Watson–Crick base pairs. We observe a strong preference for WC–WC edge interactions in this state, with almost no other edges involved (see Supporting Information). The resulting structure is therefore more extended and much more reliant on stabilizing stacking interactions than the ground state. The significant support through stacking also explains how the structure is stabilized, despite the large number of base pair mismatches. This preference for coaxial stacking is in agreement with experimental findings.^31^

Figure 5 presents the same data for the funnels containing intermediate structures. The structural variation is significant and shows stabilization of intermediates between GS and ES2 on the energy landscape. Funnel B contains structures that show very similar base pairing to the GS, but with a loss of the C–G base pair adjacent to the bulge. This loss increases the flexibility in the bulge while shortening the upper stem. In funnel C, the stem is shortened even further, but a new G–U base pair is observed. This new pairing corresponds to a slip of one position of the two needed to go from the ground state to the excited state. Funnels A and F exhibit the highest variance in the base pairing, with some nucleotides showing interactions with two nucleotides within each set of structures. Two important consequences arise from this observation. First, the base pair sliding requires the loss of multiple base pairs simultaneously. Second, the more flexible structures are no longer characterized solely by their base pairing, but by the ease with which they can switch between base pairing. Hence, a description of structural sub-ensembles purely based on base pairing is not sufficient.

**Figure 5:**
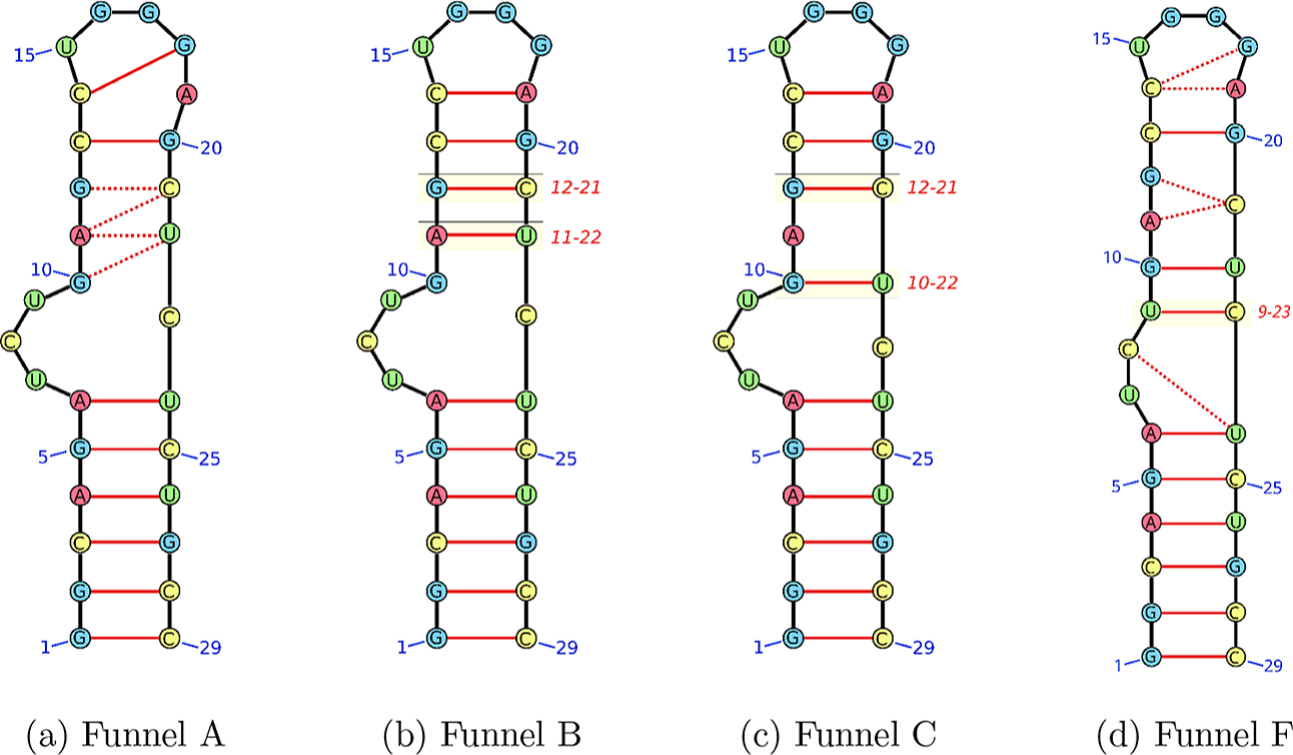
Representative schemes showing the base pairing in funnels A, B, C, and F going left to right alongside the heat maps of the nucleobase positions. Key base pairs are highlighted with boxes and labeled.

The description of the sub-ensembles by funnels from the energy landscape is very clear and a key advantage of deriving the full topography via energy landscape explorations. A more commonly adopted approach is to derive structure-based order parameters, which characterize collective variables. In an attempt to map our data in this fashion, we use a collection of geometric descriptors (see Methods for details) and use principal component analysis on all structures on the energy landscape to derive the two principal components based on structural features describing the variance in the structural ensemble. The first component explains 34.1% of the variance in the data, and the second component explains 16.7%. Figure 6 shows all minima in our database plotted against these two components, with a color scheme chosen to highlight the individual funnels described.

**Figure 6:**
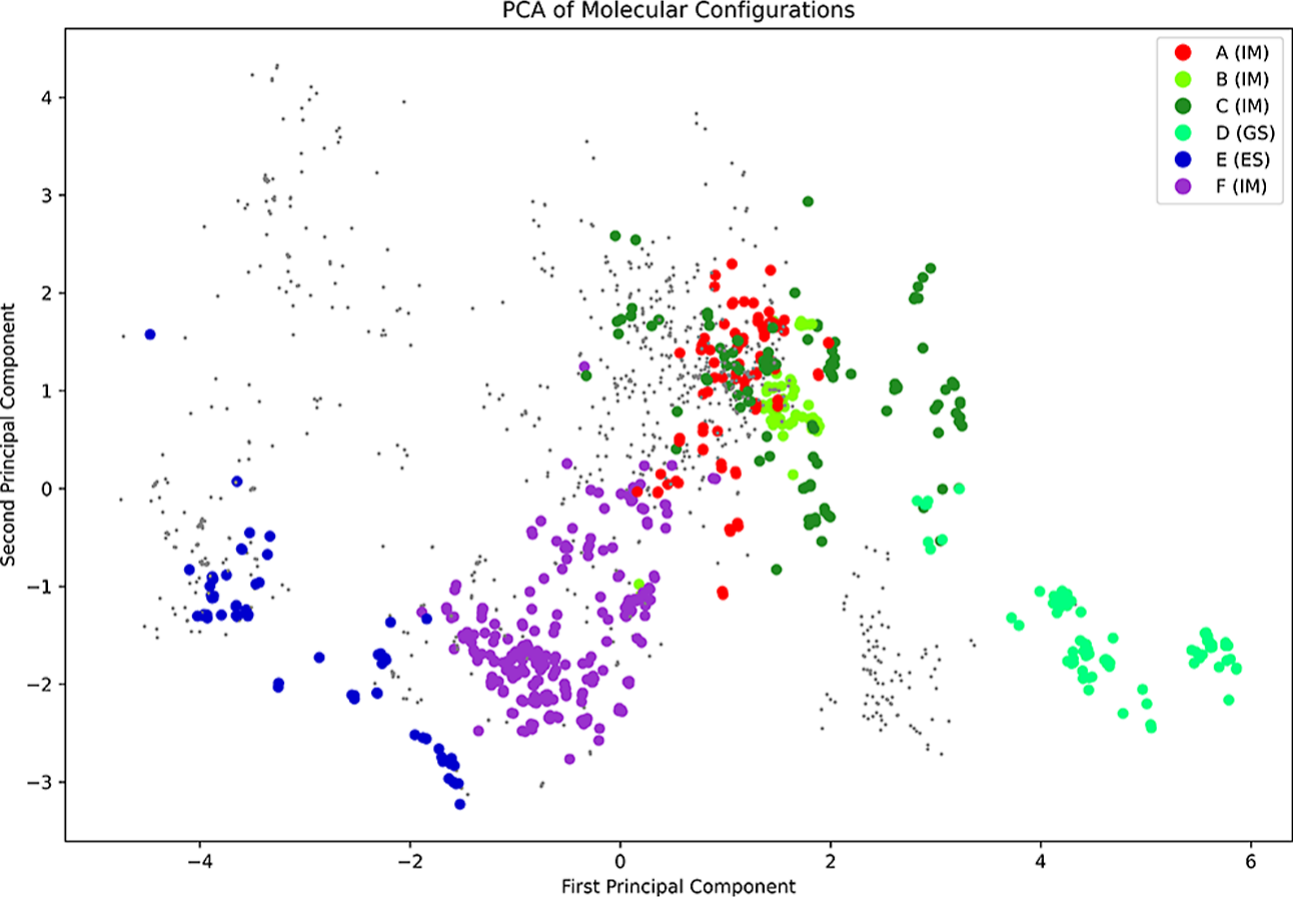
PCA results using geometric descriptors for the minima located on the energy landscape. Minima in any of the six funnels described are highlighted, while all other minima are shown as smaller dots. A clear separation between GS and ES is seen, but intermediates states are less well resolved.

The analysis clearly separates the ground state from the ES2 state. The ES2 state is clearly distinguished, while some GS structures show overlap with other funnels in this classification (in particular, with structures in funnel D). This overlap is not unexpected, given the known dynamics of the ground state and the free energy landscape changes in relative energy between funnels C and D. It is important to note how poorly such a combination of geometric features distinguishes between the intermediate states. We suggest that such descriptors, at least for RNAs, do not capture the full diversity of possible intermediates, with implications for the accuracy of sampling and analyses based on these descriptors.

#### 2.2.1 Alignment with the FARFAR-Generated Ensemble

The application of FAFAR-NMR for TAR enabled the publication of a refined structural ensemble for the ground state.^16^ Using the PCA, we can extract the same descriptors for this data set and plot them alongside the energy landscape data to identify what part of the structural ensemble is covered by both methods. From the previous analysis, we know that the EL exploration covers a large range of structures and includes a high degree of diversity in the ground state as well. The FARFAR ensemble presents a similarly diverse ensemble of refined structures, matching the experiment. The comparison will therefore give us a good understanding of how much diversity the simulations provide compared to experimentally refined data. The PCA is shown in Figure 7.

**Figure 7:**
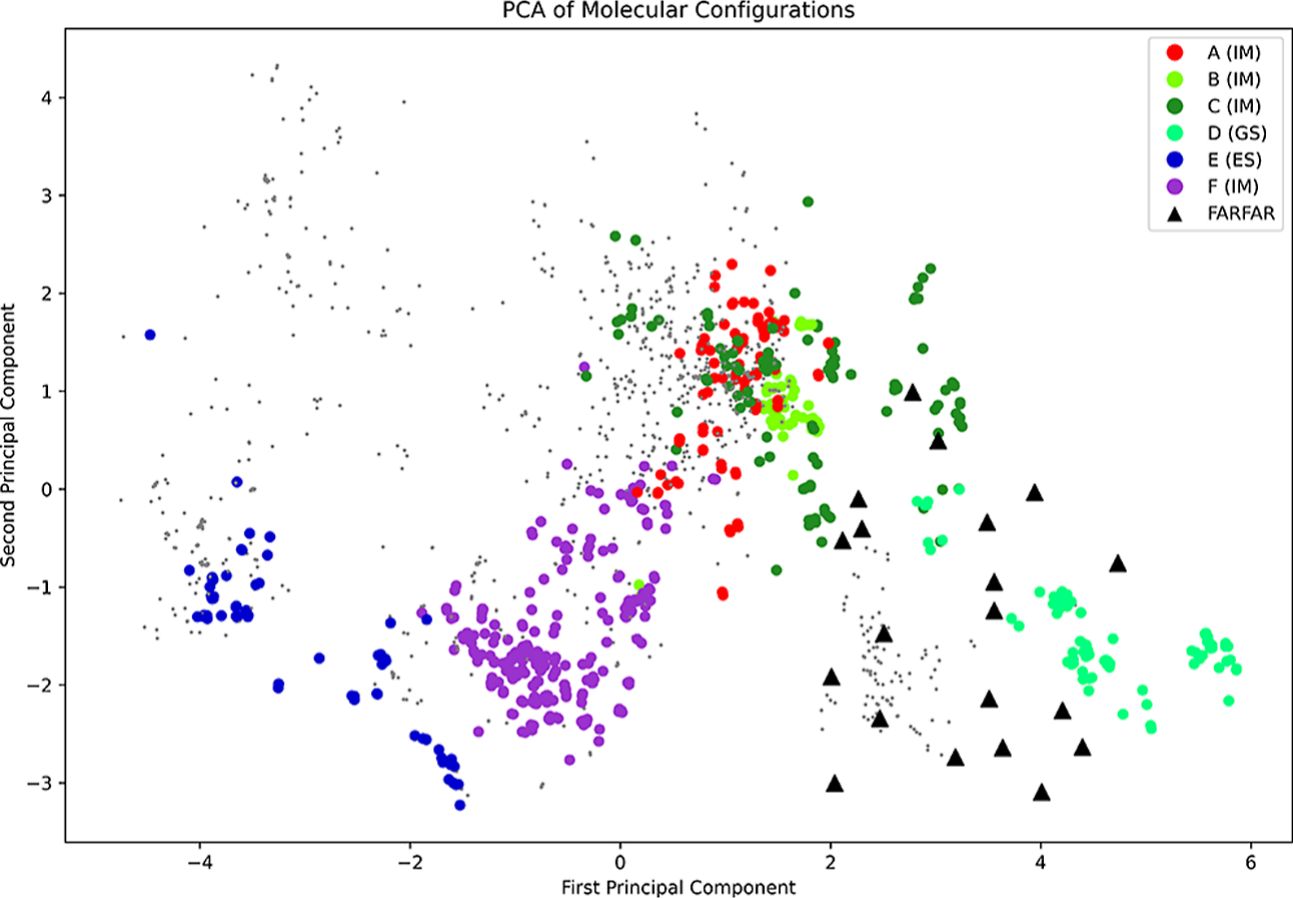
PCA of the energy landscape exploration-derived ensemble, and the aligned FARFAR-NMR ensemble shows good coverage of the ensemble of the GSs for both methods.

The FARFAR-NMR data cover a large part of the space covered by the energy landscape data for the ground state. Both methods show significant variation in these structures. This result shows us that (a) the FARFAR-NMR ensemble indeed covers a lot of the variation we observe for the GS ensemble, (b) the energy landscape sample is similarly varied, and (c) the additional insight into intermediate structures becomes apparent. There is a small shift between the structures in the FARFAR-NMR ensemble and the energy landscape ensemble for the ground state. The FARFAR ensemble also overlaps with parts of the D funnel and higher energy minima, which were not associated with a particular funnel but lie between funnels C and D. It is clear that the NMR refinement improves the local structure and represents the overall structural variations in the GS sub-ensemble. The local dynamics of the GS configurations clearly cover both structures in funnels C and D, highlighting the inherent dynamics of the bulge region, the loop dynamics, and the tendency to form a G–U wobble base pair. The purely computational approach nevertheless covers significant variance in the GS structure while also giving insights into intermediates and excited states.

#### 2.2.2 Residual Dipolar Coupling

Residual dipolar coupling (RDC) data has been used extensively to refine the local structure for TAR.^16,31,36^ While this local refinement is critical for high-quality structures, it is not clear whether it is also a sensible measure for global ensemble refinement. After computing RDCs for all minima, we repeated the PCA analysis, but this time using RDC data as features (see Methods for more details), with the results shown in Figure 8. The variance explained by the first two principal components is lower (26.3% and 18.3%) than for the structure-based analysis. Importantly, the distinct funnels are less clearly separated, and in particular, the variation between GS and ES2 is much smaller. Overall, the RDC captures the loss of base pairing in intermediate structures, which will strongly impact the RDCs, but it is less sensitive to the changes in base pairing between GS and ES2. For a given sub-ensemble, the RDCs might therefore help to refine the local structural dynamics but will be less useful to refine the entire ensemble.

**Figure 8:**
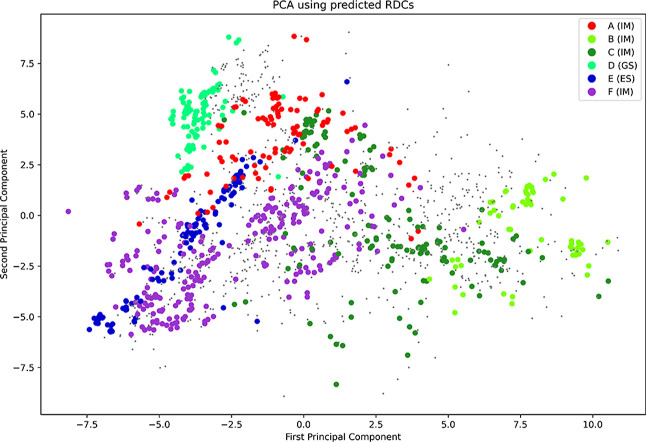
PCA analysis of all minima based on computed residual dipolar coupling. In contrast to the geometric descriptors, the funnels are not clearly separated.

### 2.3 Transition between GS and ES Configurations

The reported exchange rates of 737 *s* ^–1^ from CEST^31^ and 474 *s* ^–1^ from relaxation dispersal^31^ alongside the reported occupation probability of the ES2 allow the computation of the effective free energy barriers for the transition from GS to ES2, and vice versa. The two measurements are in agreement, yielding a barrier of 17.8 kcal/mol from GS to ES2 and a barrier of around 14.3 kcal/mol for the reverse process. A regrouping algorithm that clusters minima together allows us to obtain barriers from energy landscape exploration. The resulting barriers are 21.1 and 15.1 kcal/mol, corresponding to errors of 3.3 and 0.8 kcal/mol, respectively. Given the use of an implicit solvent and the known shortcomings in the current RNA force fields, this is a reasonable match for the predicted thermodynamics and kinetics. Importantly, it seems that the ground-state configurations are too stable, which is in line with previous findings that RNA excited states are not represented properly in RNA force fields.^10^ A potential reason might be the previously discussed mismatch in base pairing at the top of the upper stem and the overstabilization of the G–U wobble, which are likely force field artifacts.

A more interesting question is how the structures interconvert. Analyzing the pathways from the GS-like funnels to the excited state, we observe a bifurcation at the highest energy transition states between funnels A to D and E and F. On either side, we see some structures from C and F as initial intermediates. This reinforces the idea introduced earlier of stepwise changes to the base pairing.

To further understand this transition, we can consider the best (i.e., fastest) path from the ground state to the excited state and, for each minimum on this path, obtain the detailed base pairing. This analysis is shown in Figure 9.

**Figure 9:**
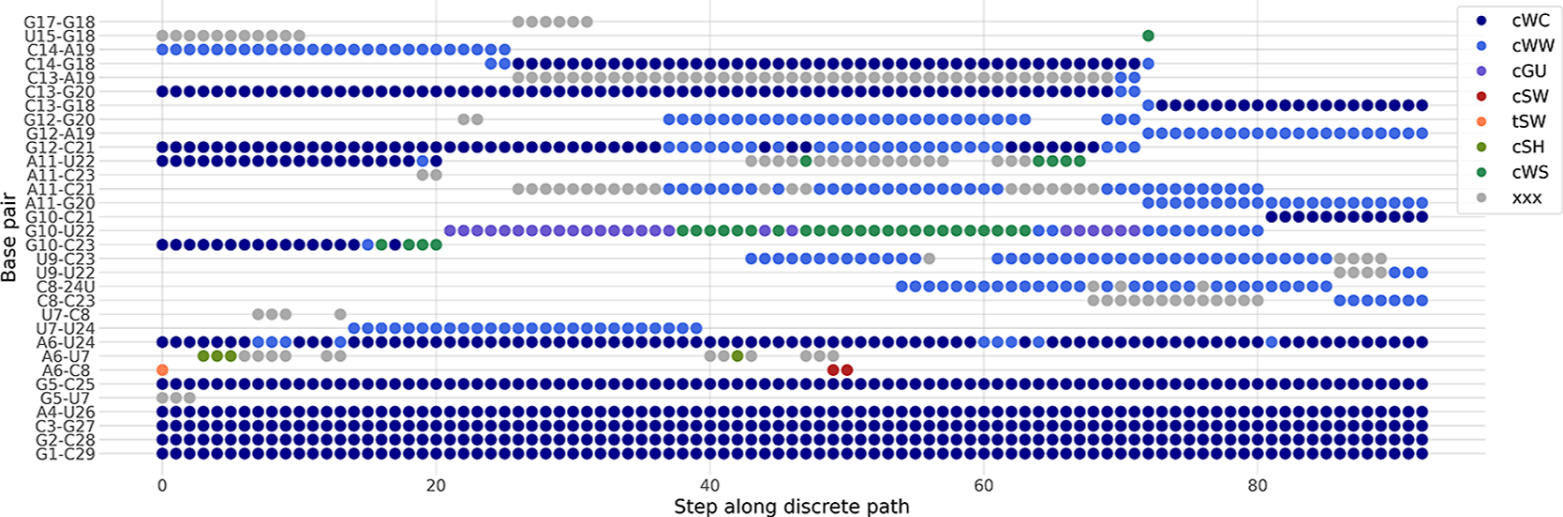
Base pairing for each minimum on the fastest path from the ground state (left) to the excited state (right). The base pairs are colored by category (according to the Leontis–Westhoff classification^37^). The classifier XXX corresponds to base pairs which fulfill the distance criteria for base pairing according to their r vectors^35^ but do not fall into a clear category of base pairs. Such likely interactions are common in simulation data. Base pairs are sequentially broken and formed, with some base pairs only appearing in intermediates. Noncanonical base pairing is key in intermediate structures.

On the left side of the figure is the GS structure, with the canonical base pairs being present in the upper stem. The main noncanonical interactions are the interaction between nucleotides at the top of the upper stem, already discussed above, and a Sugar–Watson interaction in the bulge. Going toward the excited state, we first observe interactions between U7 and the top of the lower stem forming. Around step 20 on the path, we see the loss of the two lowest canonical base pairs in the upper stem, while a new noncanonical G–U base pair is formed instead. As a result, both C23 and A11 are now unpaired. Over the next steps, these two free bases start to interact with nearby nucleotides, leading to A11–C21 and U9–C23 interactions. Rearrangements over the next steps lead to transient interactions, whereby the G10–U22 base pair adopts different conformations, likely due to the motions of the bases in the bulge. At the same time, base pairing adjacent to the loop is changing, with the formation of the C14–G18 canonical base pair. This change in turn allows for the interactions of now unpaired A19. These changes, both below and above the G12–C21 and C13–G20 base pairs, lead to formation of new interactions between G12 and G20, while the WC base pairs are maintained. At this stage, all base pairs in the upper stem are primed for a slippage, and this event occurs around step 70, when the C13–G18, G12–A19 and A11–G20 base pairs, found in the ES, are formed. This change in the upper part of the stem leads to the breaking of the persistent G10–U22 base pair and the formation of the second WC base pair, G10–C21, found in the ES upper stem. The remaining steps require reshuffling of the lower part of the stem to form the final base pairs.

Two important features emerge. G10 moves from one canonical base pair in the ground state to another in the excited state. This movement requires the slippage of two nucleotides in the opposing strand. This movement occurs stepwise, with a strong G–U base pair formed as an intermediate. This base pair anchors the lower part of the stem, facilitating the motion of adjacent nucleotides. Likely, mutations of this nucleotide will significantly impact the transition mechanism and kinetics.

Second, the main base pair changes happen in cascades (around steps 20 and 70), where multiple base pairs are formed or broken. The picture indicates that local dynamics allow the RNA to reach structures where multiple changes can occur simultaneously such that the loss of base pairs is compensated, at least in part, by the formation of new base pairs.

### 2.4 Identification of Potential Binding Sites

Given the extensive set of structures describing the structural ensemble, another question that can be considered here is the existence of a difference in the interaction patterns of the different states. First, such differences are key to binding to other biomolecules, as suggested for the ability of the GS and ES2 to bind Tat and Cyclin T1.^31^

A second set of interactions to consider is with small molecules. Various small-molecule binders have been proposed for this stemloop.^39^ It is well known that TAR can bind to both small, cyclic peptides^40^ and small molecules.^41^ Using statistical interaction fields (SMIFs),^38^ we searched for putative interaction sites. Our main aim with this search was to characterize the difference in binding sites between the GS and ES2, not to identify new potential binding pockets. The binding pocket that is known to interact with small peptides is roughly of similar size in both structures, as shown in Figure 10a, but otherwise they are rather different. For the ground state, the extended bulge enables stacking interactions, as well as a large patch for both hydrophobic and electrostatic interactions. In contract, for the ES, we only see the latter, and they are shifted upward, likely due to the coaxial stacking that extends the stemloop. The ensemble derived from energy landscape explorations can therefore be used to identify putative differential binding sites and modes for further validation in the future. An incidental finding of this analysis was the existence of a hole in the RNA surface for structures in funnels A, B and C. An example is shown in Figure 10b. While we are not aware of a small ligand binding to such a structure having been described in the literature, a configuration with a much more embedded small ligand is known.^42^ These structures form part of the transition path from GS to ES2 and therefore may provide a novel structural target for small-molecule binders.

**Figure 10:**
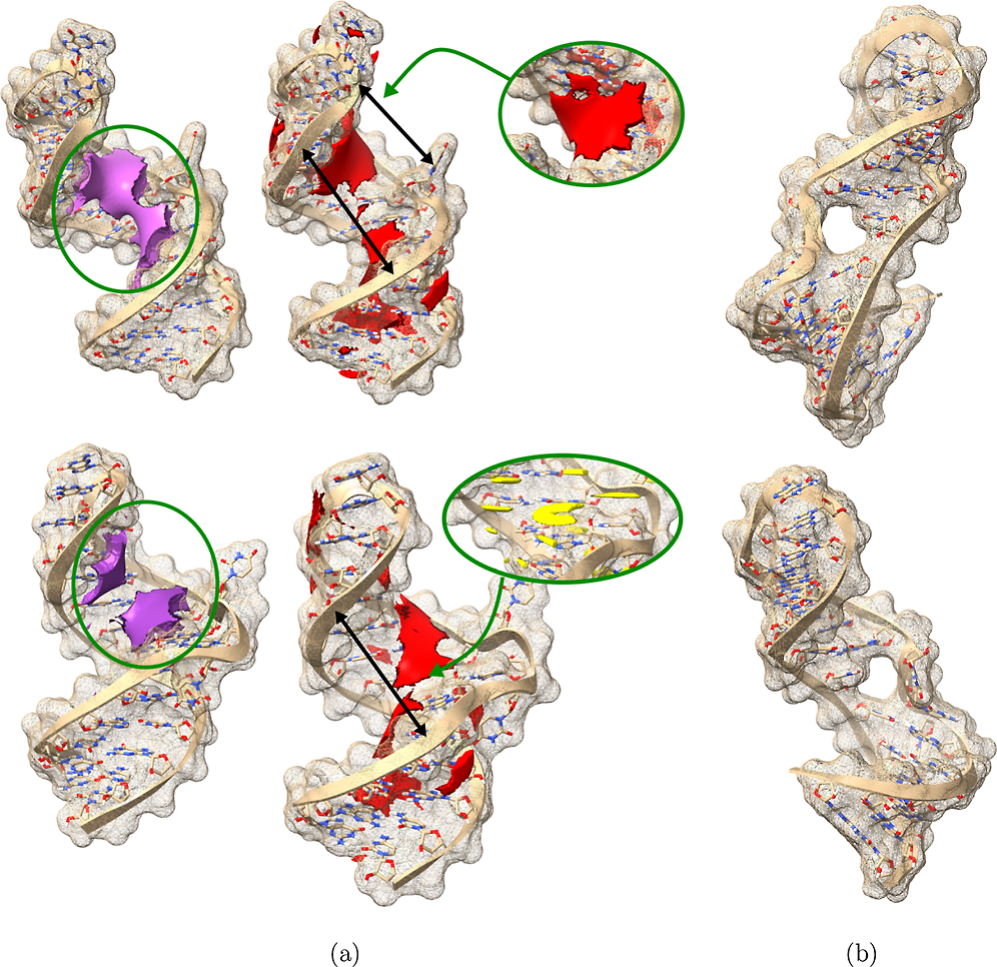
Illustration of putative binding sites. (a) Statistical interaction fields^38^ for the ES2 state (top two structures) and ground state (bottom two structures). Electrostatic fields are shown in purple, hydrophobic fields in red, and stacking fields in yellow. A shift in the position of the electrostatic field is highlighted (green circle on the left). The opening of the groove, as shown by black arrows, is broadly conserved between 14.0 and 15.9 Å. The ES2 structure shows a larger hydrophobic patch at the back of the loop (shown as insert), and the GS shows the ability to form stacking interactions (insert bottom). (b) A hole in the surface located in intermediate structures, opening up in the bulge region.

### 2.5 The Energy Landscape of the ES2 Variant

Geng et al.^31^ introduced a variant of the TAR stemloop, where they extended the loop region to introduce more base pairing in the excited state, thereby stabilizing this state for characterization. In addition to the canonical TAR stemloop, we also analyzed the energy landscape for this mutant. The disconnectivity graph for this variant is shown in Figure 11.

**Figure 11:**
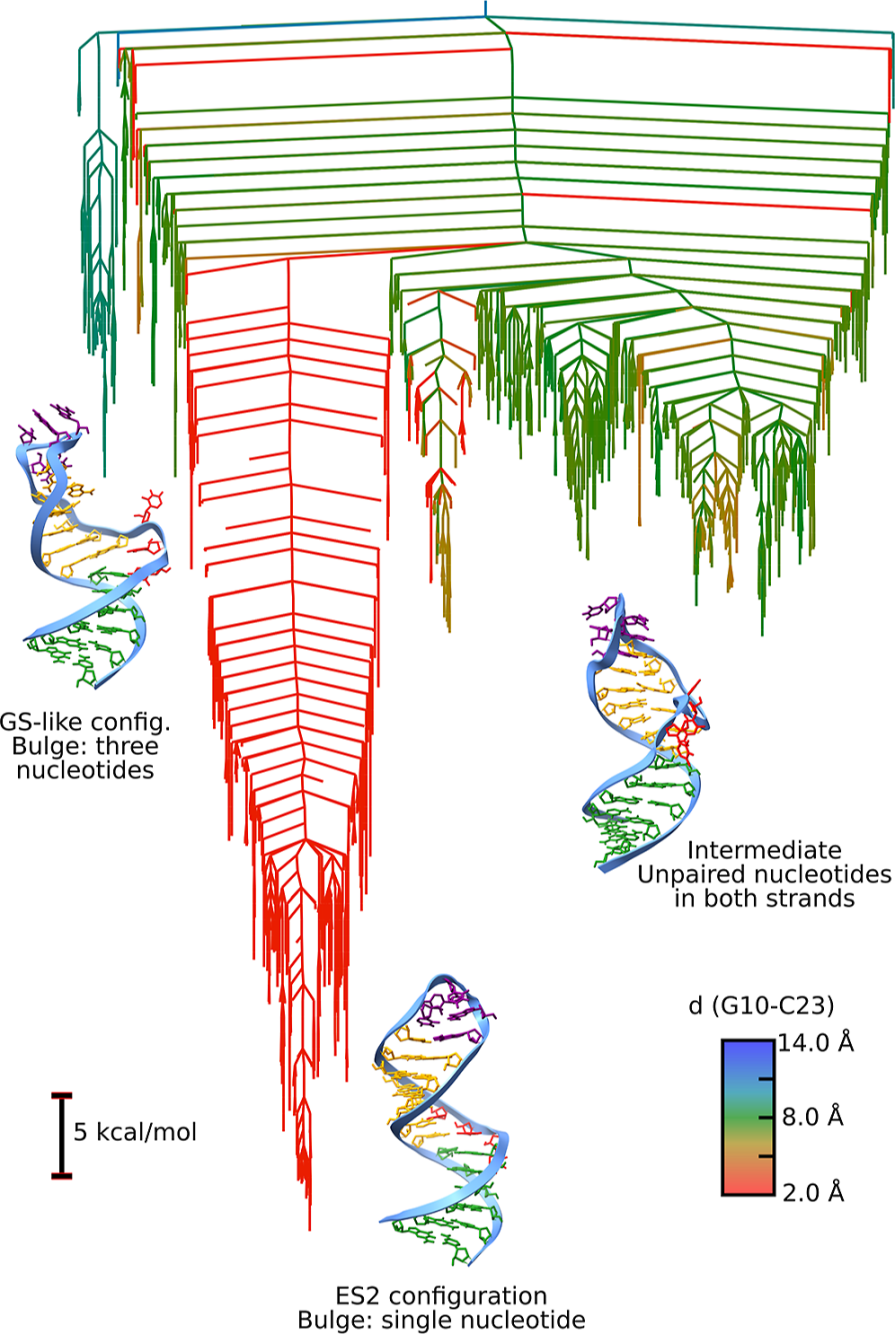
Potential energy landscape for the ES2 variant as reported by Geng et al.^31^ The ES configuration is the ground state, while the GS-like configuration is significantly destabilized. The intermediate structures are also less populated.

The energy landscape is significantly different from the width-type sequence. The additional base pair in the ES-like configuration stabilizes this structure significantly, while the relative stability of the GS-like structure is gone. The observed upper stem in the ES-like state is formed by eight base pairs, while the GS-like state has only five base pairs. The intermediate structural variety is also significantly depleted. Free energy barriers reflect this difference with estimated barriers from ES to GS at around 41.1 and 11.6 kcal/mol in the opposite direction. Two key observations emerge from this analysis. First, the single mutation significantly impacts the entire landscape, and the stabilization of the ES-like state is not equivalent to a reversal of the properties of the TAR stemloop. Second, the change also depleted the variety of intermediate structures, highlighting how the transition mechanism is also optimized in the WT sequence. RNA engineering therefore requires such considerations of the RNA dynamics and pathways rather than more readily accessible structural features.

## 3 Conclusion

For this work, we set out four criteria to assess the ability of computational energy landscape explorations to provide insight into RNA structural ensembles with a computing-only strategy. Overall, the energy landscape framework proves to give useful data for each of the criteria, highlighting the importance of sampling.

We were able to reproduce the correct thermodynamic stability, key structural features, and time scales of transitions between GS and ES2 for the TAR stemloop. While small deviations from the experiment are found, the predicted free energy barriers closely match the experiment. We believe that the energy landscape framework can overcome the sampling problems in characterizing RNA structural ensembles, and despite the use of an implicit solvent in this context, we still report good agreement with the experiment.

Local refinement via experimental constraints can improve this sampling further, but such refinements do not necessarily improve the global ensemble properties. This result is not a surprise, given that intermediate structures are short-lived and, hence, will not produce clear signatures in experimental data.

The additional insight from this work is also related to these short-lived intermediates. First, characterization of the transition path reveals key interactions in these structures, such as the G–U base pair stabilizing structures. Second, the analysis can reveal potential binding sites for small molecules, in both stable structures and intermediates. Finally, the comparison with the ES2 variant shows how delicate the balance between interactions is between ground and excited states and that manipulation of RNA dynamics is much more intricate than the stabilization of target structures.

## 4 Methods

### 4.1 Energy Landscape Explorations

Energy landscape explorations^18,19^ with discrete path sampling^20,21^ effectively applies a coarse graining scheme to the potential energy landscape. All local minima are retained in this description alongside the transition states, here formally defined as Hessian-index-1 saddle points. The local minima are connected by these transition states, resulting in a graph representation of the energy landscape, i.e., a kinetic transition network (KTN).^43,44^ This representation faithfully conserves the thermodynamic and kinetic properties of the molecular system (assuming appropriate sampling) alongside the structural features for each minimum. The key feature of this approach is the geometric definition of the minima and transition states, meaning that all explorations are independent of the barrier height, overcoming the key limitation of other simulation methods while retaining dimensionality.

The main challenge of this approach is to identify transition state candidates that connect parts of the energy landscape. To this end, we employed the quasi-continuous interpolation (QCI) scheme^45,46^ to build physically relevant interpolation bands between distant structures. These interpolation bands were then optimized using the doubly nudged elastic band (DNEB) algorithm.^47–49^ Energy maxima on the resulting interpolation were considered as transition state candidates and converged to true transition states with hybrid eigenvector-following (HEF).^50^ Associated minima for each transition state were located using the approximate steepest-descent paths along the unique eigendirection.

Specific sampling schemes for removal of kinetic traps,^51^ short cutting of high barriers,^52^ and enhanced local sampling were used to converge the exploration.^53^ Convergence was assumed when the heat capacity curve and the observed landscape topography no longer changed with further sampling.

Free energies are computed by using a harmonic superposition approach by which the overall partition function is determined by using weighted contributions from individual stationary points. The vibrational degrees of freedom are treated as harmonic, introducing a small error. The good match between the experimental and computed free energies shows the feasibility of this approach. Convergence of the sampling as indicated above corresponds to converging the density of states for the relevant transitions. The approach works as long as we do not observe one-to-many transitions, such as complete unfolding and refolding. Both our findings and the experimental data do not suggest such a process.

### 4.2 Force Field and Starting Points

The AMBER ff99 force field with the bsc0 α/γ corrections^54^ and the χ OL3 modifications^55,56^ with an implicit solvent (igb2)^57^ was used for this study. This combination of the force field and the solvent model yields reasonable energy landscapes compared to alternatives^10^ and has been used for similar work^22,23^ that agreed with experimental data.

For this work, three starting structures were used: one structure reported for the ground states for TAR (pdb: 7JU1) and the ES2 mutant (pdb: 8U3M) and two configurations derived from secondary structures. For these structures, we used the secondary structure for the ground state and excited state for both systems and created a corresponding 3D structure with RNAcomposer.^58,59^ All three structures for the two systems were minimized in energy to obtain local minima, which were then used as starting points to explore the energy landscape.

Labeling of nucleotides in this work starts at 1, shifting the annotations relative to other reported work. Position 1 corresponds to G17 in the full RNA, with the bulge nucleotides U7, C8, and U9 in our notation corresponding to U23, C24, and U25, respectively.

### 4.3 Analysis

Energy landscapes are presented as disconnectivity graphs,^32,33^ using the distance between the center of the nucleobases for G10 and C21 as the order parameter for coloring. The distance is computed by using the nitrogen atoms in the Watson–Crick edge (N1 for A and G and N3 for C and U). The choice of this order parameter was based on the sequential nature of changes to base pairing, with interactions of G10 observed with C21, U22, and G23, distinguishing clearly between the ground state, excited state, and intermediates.

Funnels were identified manually, using key branches as a guide for cutting points. The analysis included all funnels with low energy structures and the funnels of significant size and depth at higher energies.

The geometry-based descriptors for the PCA are the distance between all base pairs highlighted in Figures 4 and 5 (8–23, 9–23, 10–21, 10–22, 10–23, 11–22, 12–21), the puckering angle for residues A6 to U9, the Euclidean angles for the helix–bulge–helix as described here,^60^ and the solvent-accessible surface for nucleotide 23. The base pair distances and solvent-accessible surface area were chosen based on the features of the distinct states observed in the different intermediate structures. The puckering for the bulge nucleotides and Euclidean angles have been suggested by others as good descriptors of the transition.^16,31^ These features were computed using cpptraj.^61^ All features were scaled before PCA was conducted. The relative importance of these features to the principal components is provided in the Supporting Information. RDCs were computed using PALES^62^ for each minimum. All RDCs were used as features for the PCA conducted.

Base pairing information was obtained using Barnaba.^35^

## Supplementary Material



## Data Availability

The databases, examples structures, and analysis files are available on zenodo: DOI: 10.5281/zenodo.17674529.

## Notes

PCA feature importance, hierarchical clustering for geometry-based and RDC-based feature sets, disconnectivity graphs colored by the geometric features, heat maps for relative base pair orientations in the funnels, and histograms showing the distributions of key characteristics in the energy landscape database versus the ANTON and FARFAR ensembles (PDF)
