# Supplementary material for: Decoding RNA Structural Ensembles: Energy Landscape Exploration of the TAR Stemloop
Source: J Chem Theory Comput. 2026 Jan 5;22(2):1111–21. doi: 10.1021/acs.jctc.5c01677 (PMC12854738; doi:10.1021/acs.jctc.5c01677)
Supplement: Supplementary file 1 [file ct5c01677_si_001.pdf]

Supporting Material: Decoding RNA Structural Ensembles: Energy Landscape Exploration of the TAR stemloop

Konstantin Röder*

Randall Centre for Cell and Molecular Biophysics, King's College London, London

E-mail: konstantin.roeder@kcl.ac.uk

S1 Feature importance for PCA

Figures S1 and S2 show the feature importance for the principal component analysis for the analysis of all minima and the minima in the funnels, respectively.

S2 Hierachical clustering

To analyse the ability to distinguish between structures globally using geometric parameters and RDC data, we used hierarchical clustering. The results are shown in Fig. S3 and S4, respectively.

S3 Geometric order parameters

Fig. S5 and S6 show how eight of the geometric parameters distinguish between different funnels, when sued to colour disconnectivity grraphs.

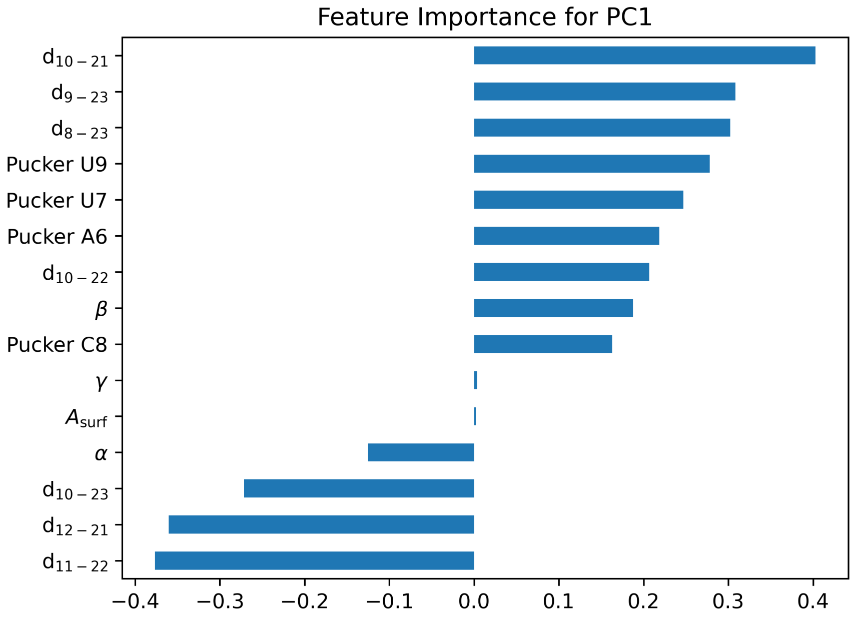

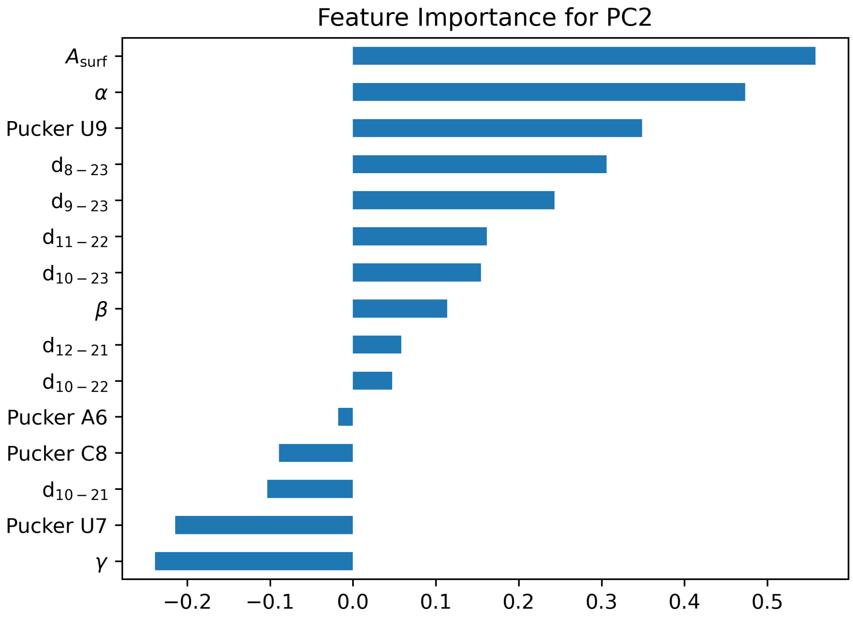

Figure S1: PCA feature importance for for using geometric descriptors for all minima.

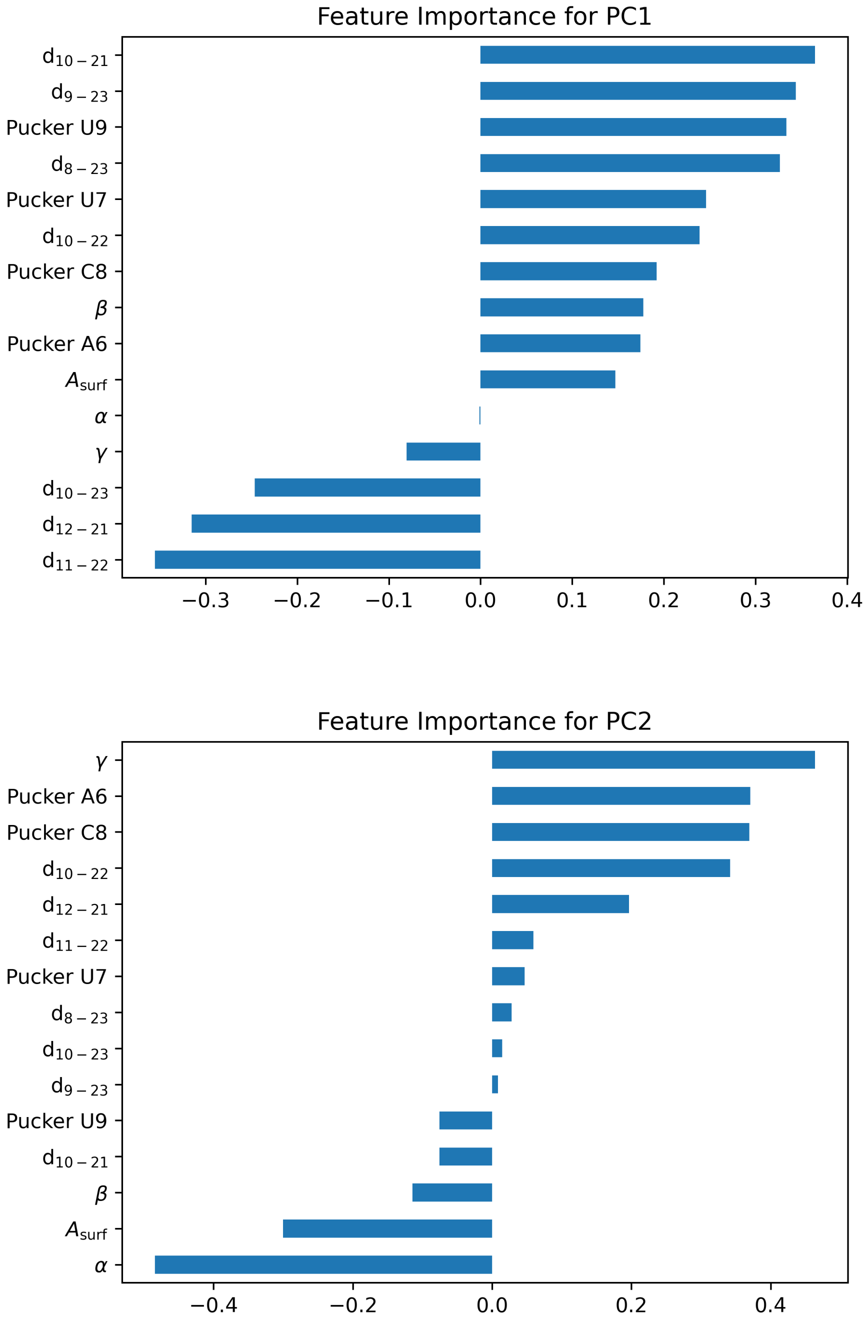

Figure S2: PCA feature importance for for using geometric descriptors for minima in the funnels A to F.

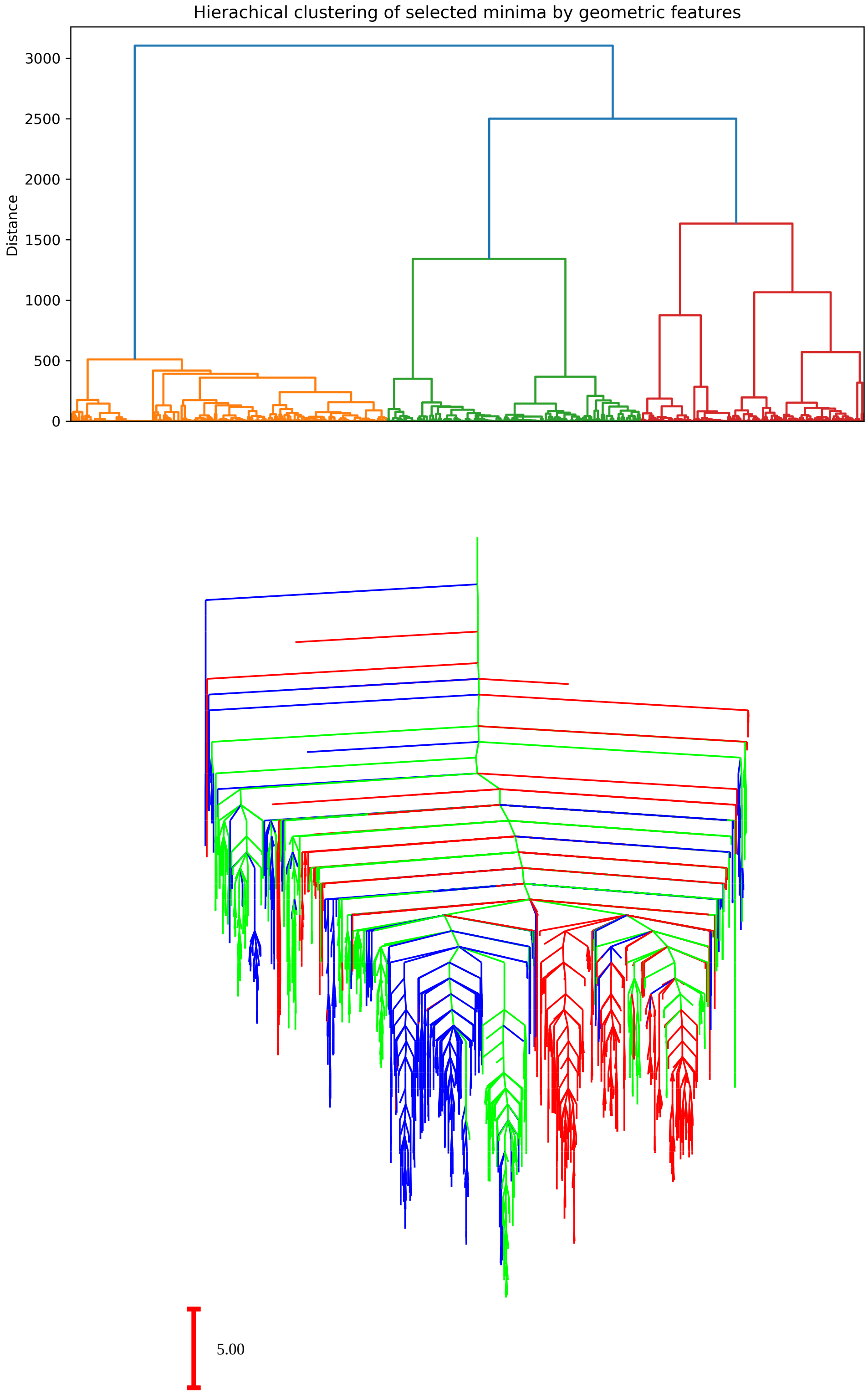

Figure S3: Hierarchical clustering (top) and associated colouring of the disconnectivity graph (bottom) using geometric observables for every minimum.

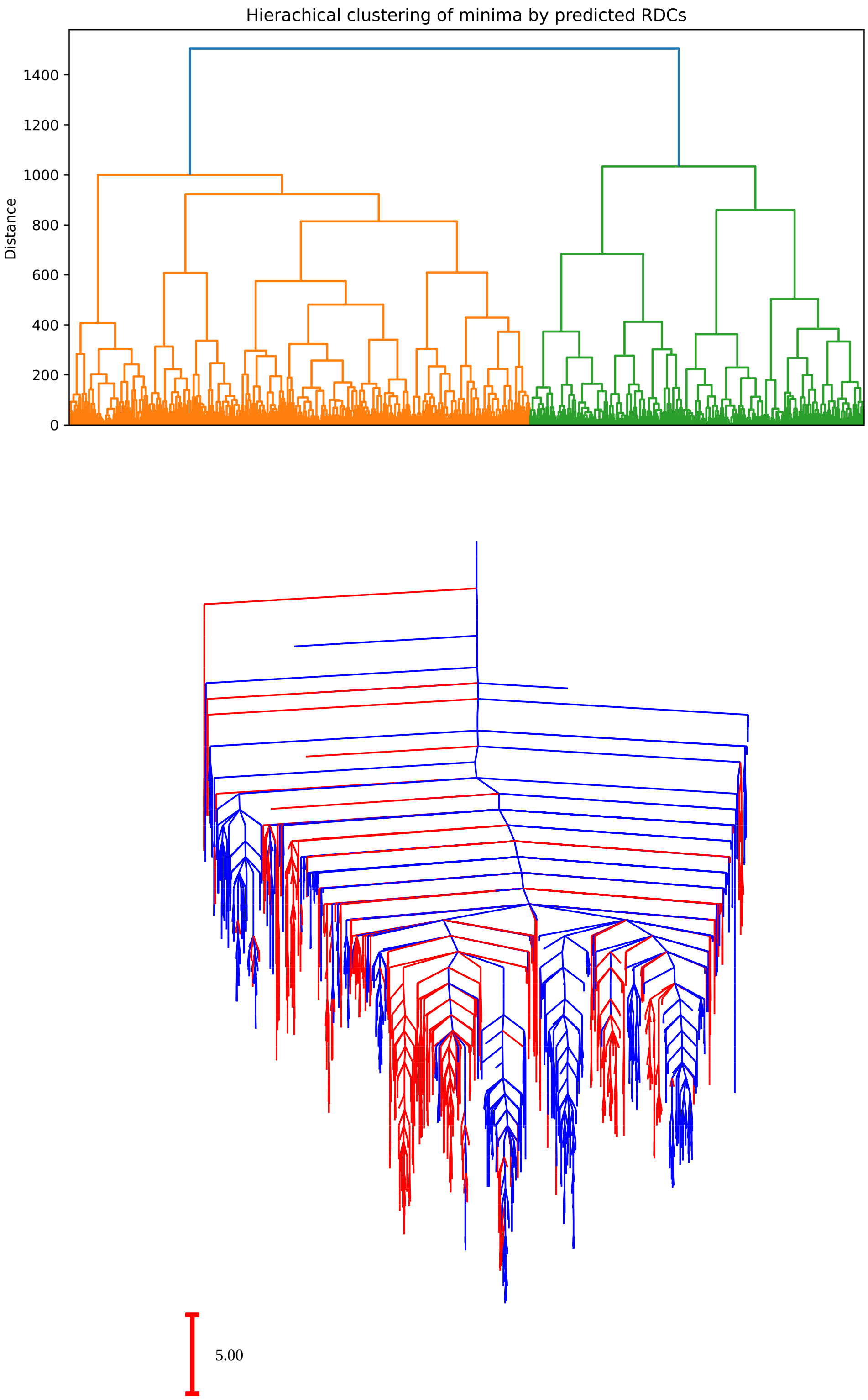

Figure S4: Hierarchical clustering (top) and associated colouring of the disconnectivity graph (bottom) using RDC data for every minimum.

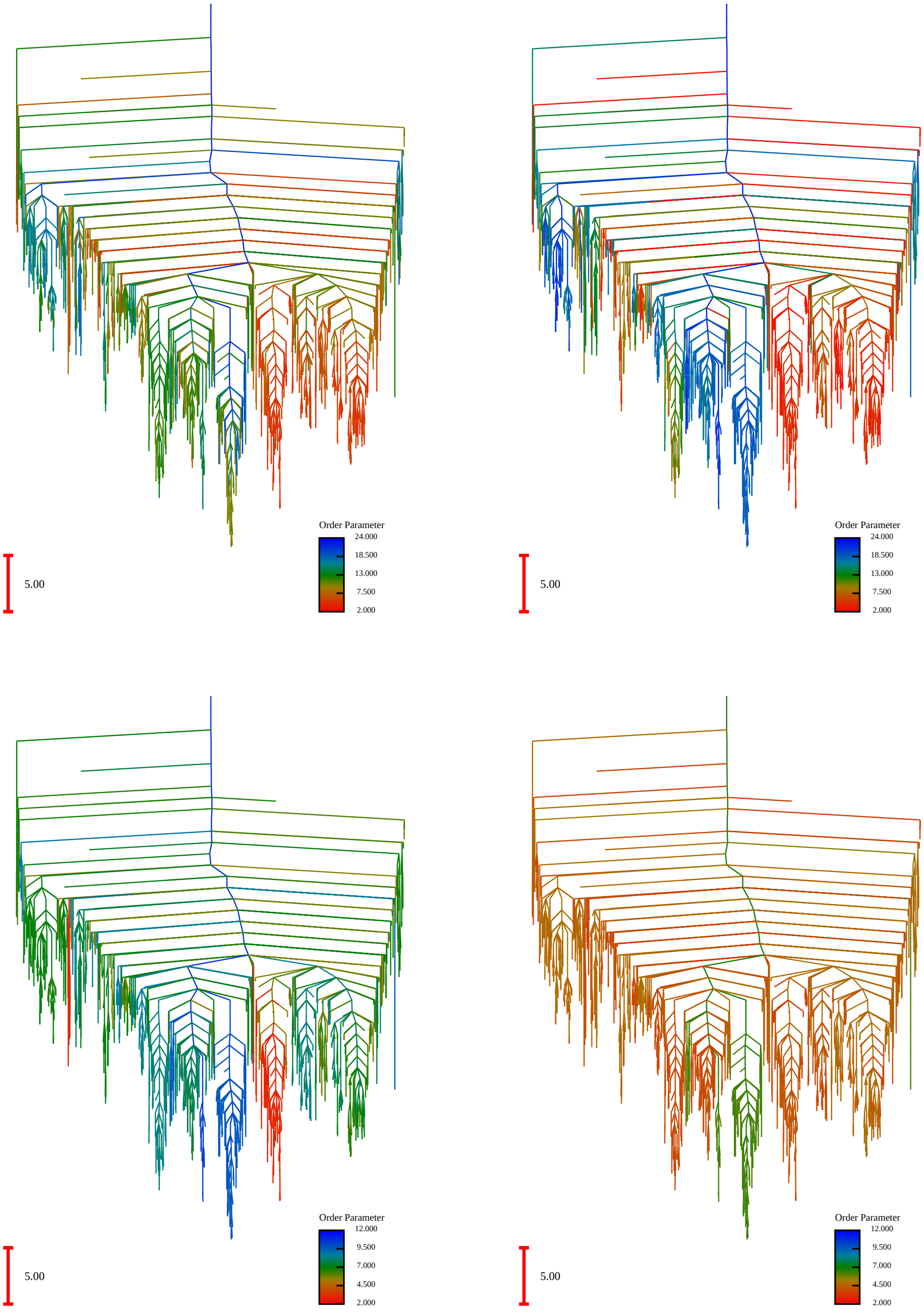

Figure S5: Potential energy disconnectivity graph coloured by the distance between C8 and C23 (top left), between U9 and C23 (top right), between G10 and C21 (bottom left), and between G10 and U22 (bottom right). Shorter distances are read, longer ones in blue.

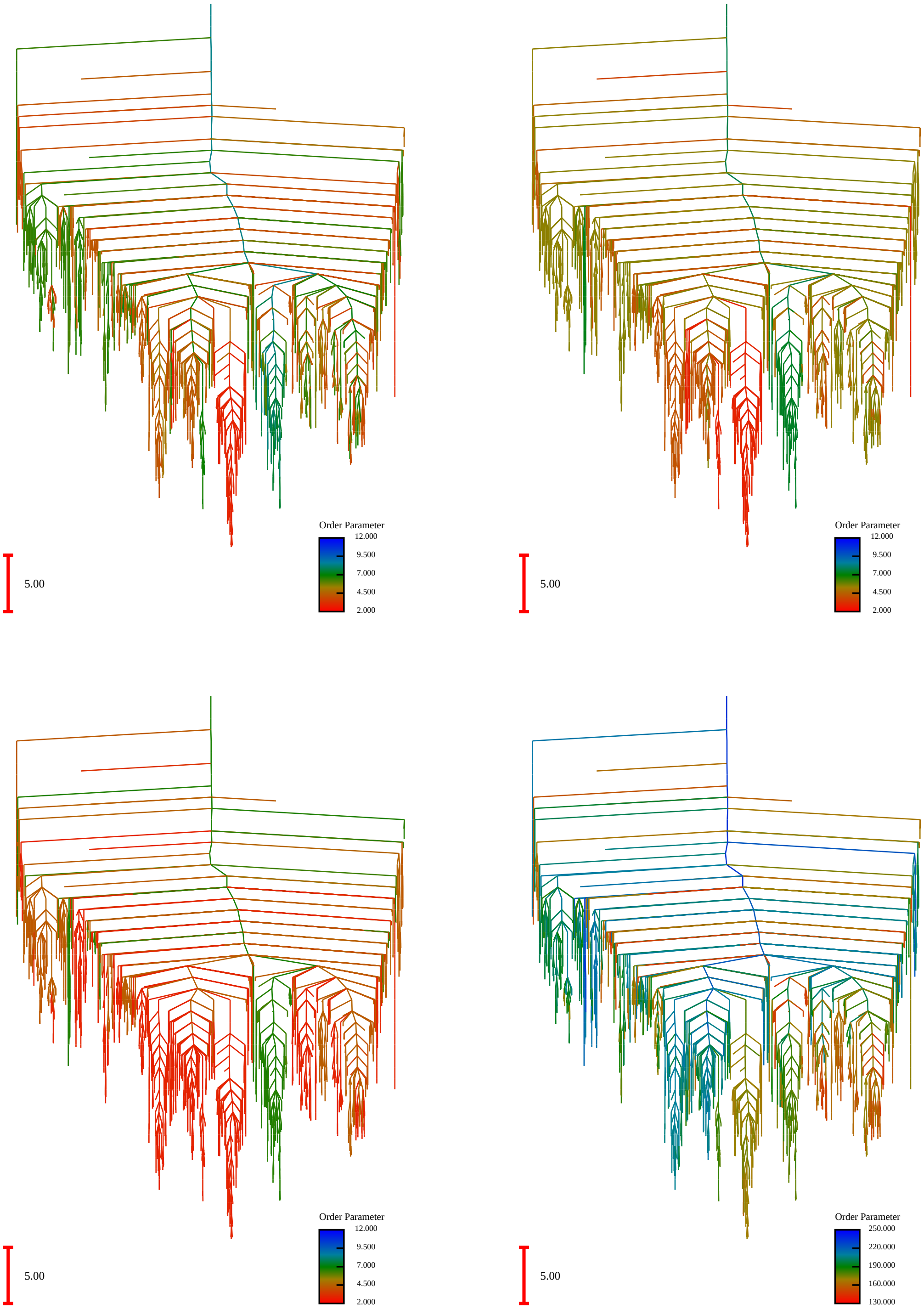

Figure S6: Potential energy disconnectivity graph coloured by the distance between G10 and C23 (top left), between A11 and U22 (top right), between G12 and C21 (bottom left), and the solvent exposed surface forming the hole in the structure (bottom right). Shorter distances are read, longer ones in blue.

S4 Relative base orientation in individual funnels

Fig. S7 shows heat maps for the relative orientation of base with respect to each other. In the ES2 funnel, we observe mainly WC-WC edge interactions, while more non-canonical interactions are observed in other funnels, clearly highlighting the increased coaxial stacking in the excited state.

S5 Comparison of the EL data with the FARFAR and ANTON ensembles

To illustrate the match between FARFAR and ANTON data and our landscape exploration results, Fig. S8 to S12 show comparisons of the distribution of a set of geometric descriptors for the three data sets.

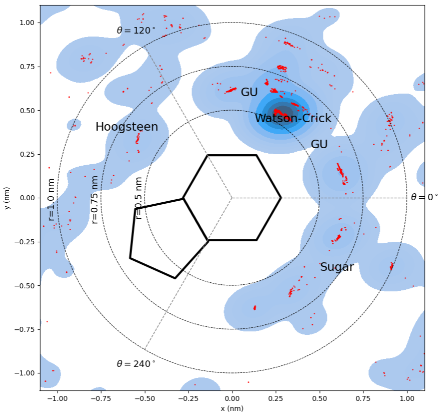

Funnel A

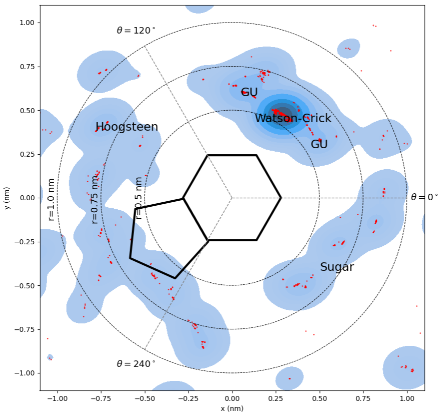

Funnel B

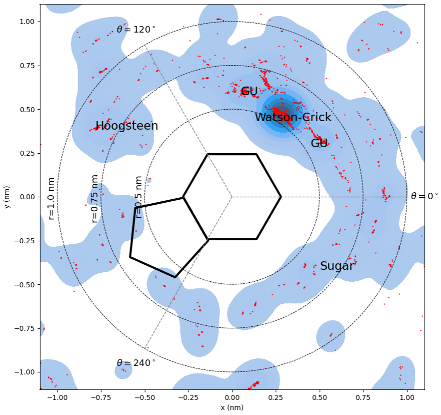

Funnel C

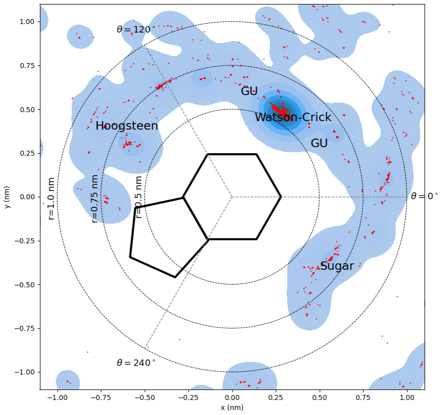

Funnel D

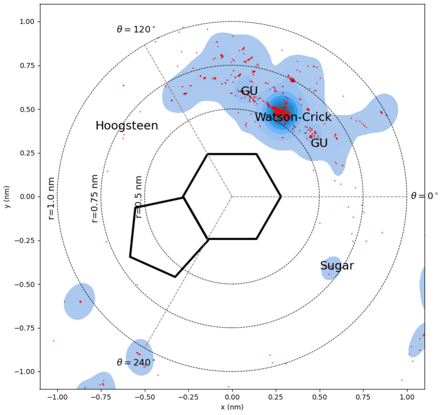

Funnel E

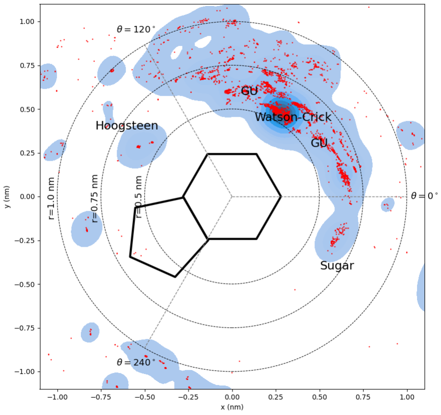

Funnel F

Figure S7: Heat maps for the relative orientation of bases in base pairing for each funnel derived using barnaba and the code on the barnaba git.

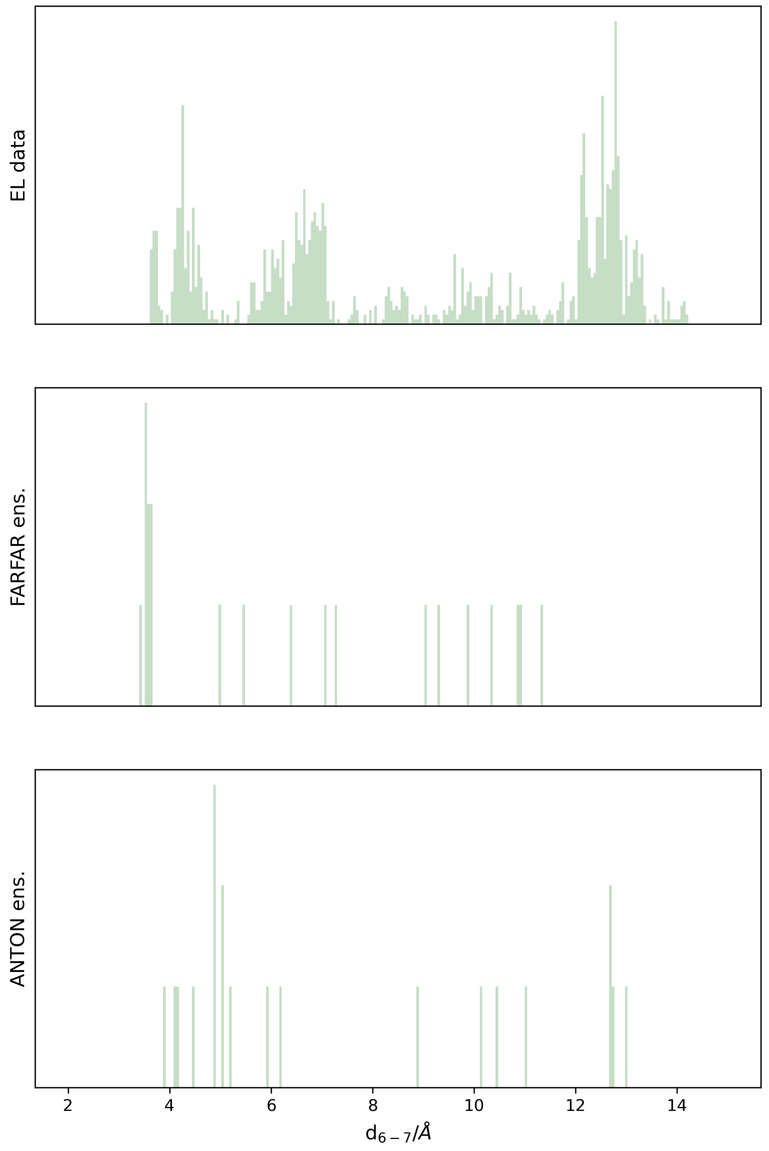

Figure S8: Histograms of the distance between A6 and U7, showing the bulge dynamics.

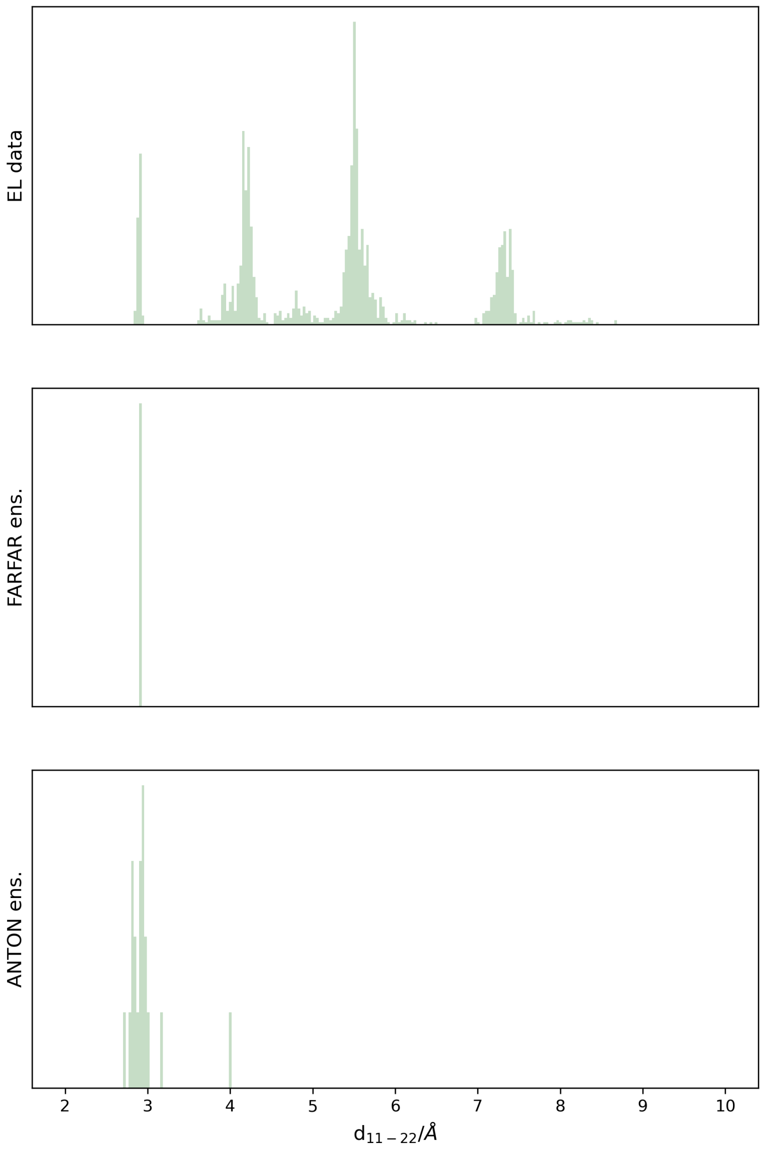

Figure S9: Histograms of the distance between A11 and U22, a canonical base pair formed in the ground state.

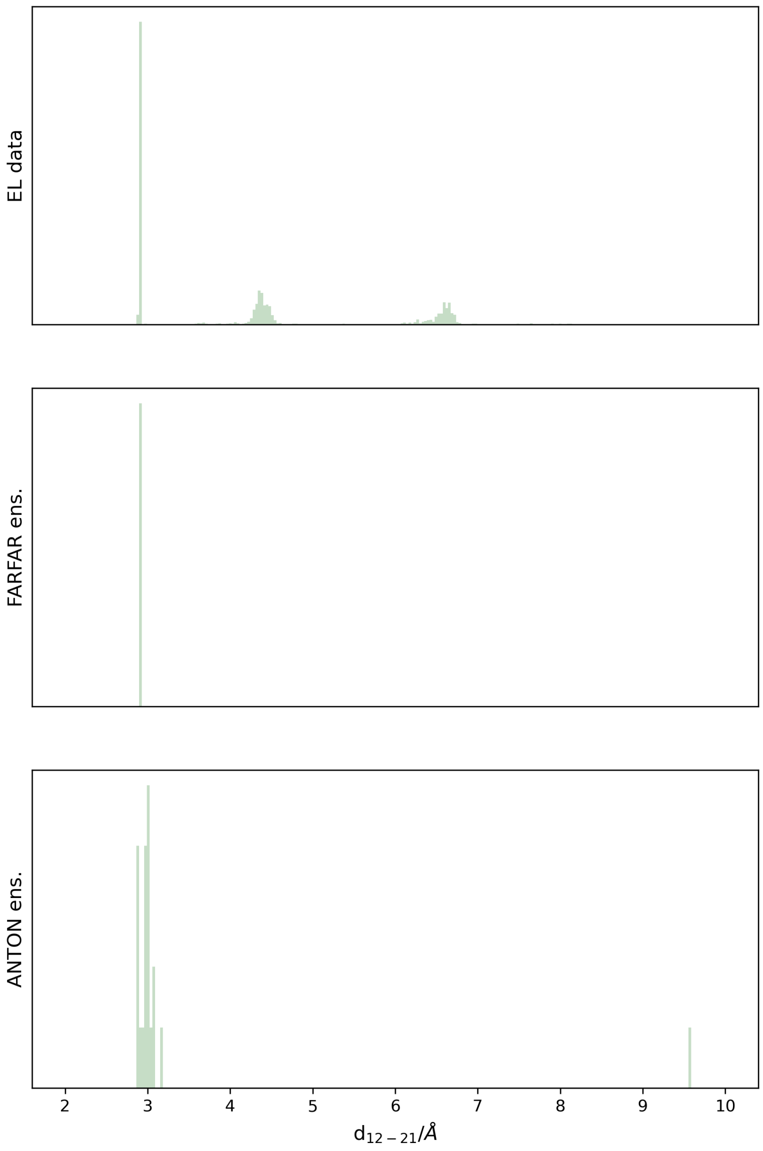

Figure S10: Histograms of the distance between G12 and C21, a canonical base pair formed in the ground state.

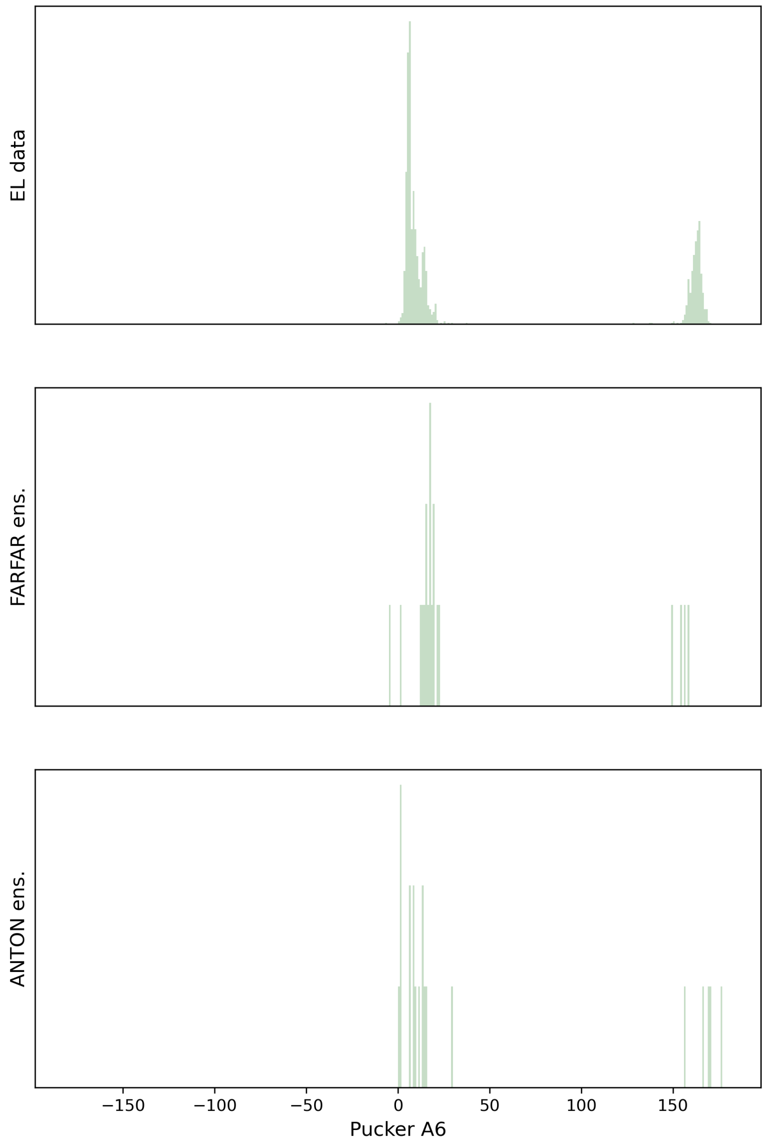

Figure S11: Histograms of the sugar pucker dihedral angle in A6.

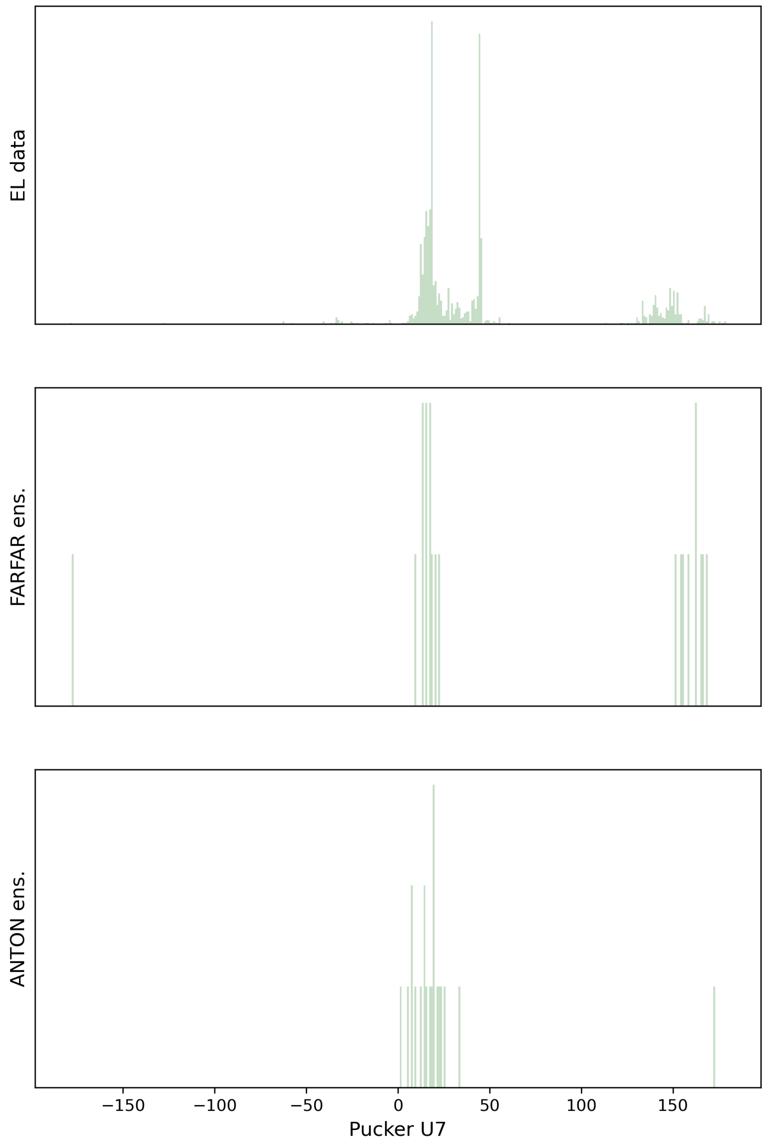

Figure S12: Histograms of the sugar pucker dihedral angle in U7.
